# Synthetic cannabinoid receptor agonists inhibit the cardiac voltage-gated potassium channel hERG

**DOI:** 10.1038/s41401-026-01876-9

**Published:** 2026-07-21

**Authors:** Nina E. Ottosson, Damon J. A. Frampton, Tanadet Pipatpolkai, Caitlyn Norman, Urban Karlsson, Amaia Jauregi-Miguel, Maryke Venter, Akshay Sridhar, H. Peter Larsson, Henrik Gréen, Sara I. Liin

**Affiliations:** 1 Department of Biomedical and Clinical Sciences, Linköping University, Linköping, Sweden; 2 Chemical Biology Consortium Sweden, Science for Life Laboratories (SciLifeLab), Linköping University, Linköping, Sweden; 3 School of Physical and Mathematical Sciences, Nanyang Technological University, Singapore, Singapore; 4 Department of Forensic Genetics and Forensic Toxicology, National Board of Forensic Medicine, Linköping, Sweden

**Keywords:** arrhythmia, electrophysiology, KCNQ1, molecular dynamics

## Abstract

Synthetic cannabinoid receptor agonists (SCRAs) are a large group of structurally diverse designer drugs (analogues of controlled substances) associated with intense and sometimes fatal intoxication. Cardiac symptoms, including tachycardia and arrhythmia, are common consequences of SCRA consumption. However, little is known about the mechanisms through which SCRAs may perturb cardiac rhythm. Here, we used electrophysiological techniques to screen 36 SCRAs on two ion channels responsible for cardiomyocyte repolarization, hERG (also called K_V_11.1) and K_V_7.1/KCNE1. We report that the majority of tested SCRAs inhibited hERG, primarily by reducing channel conductance, and some also inhibited K_V_7.1/KCNE1. In silico data suggest that SCRAs may use both a known drug binding site in the central cavity of the hERG channel, shared by established hERG blockers like astemizole, and a recently identified side pocket site. Experimental and in silico data suggest SCRA structural features associated with prominent inhibitory effects on hERG, with chemical moieties allowing bond formation and/or the ability to fit into binding sites being important. Structure-activity relationships (SAR) for SCRA effects on hERG, K_V_7.1/KCNE1 and the cannabinoid receptor 1 (CB_1_) varied, demonstrating the need to assess SCRA effects on multiple potential targets. In conclusion, we found SCRAs to be inhibitors of cardiac voltage-gated potassium channels important for cardiomyocyte repolarization, highlighting the importance of more extensive investigation of SCRAs on cardiac function.

## Introduction

**Fig. 1:**
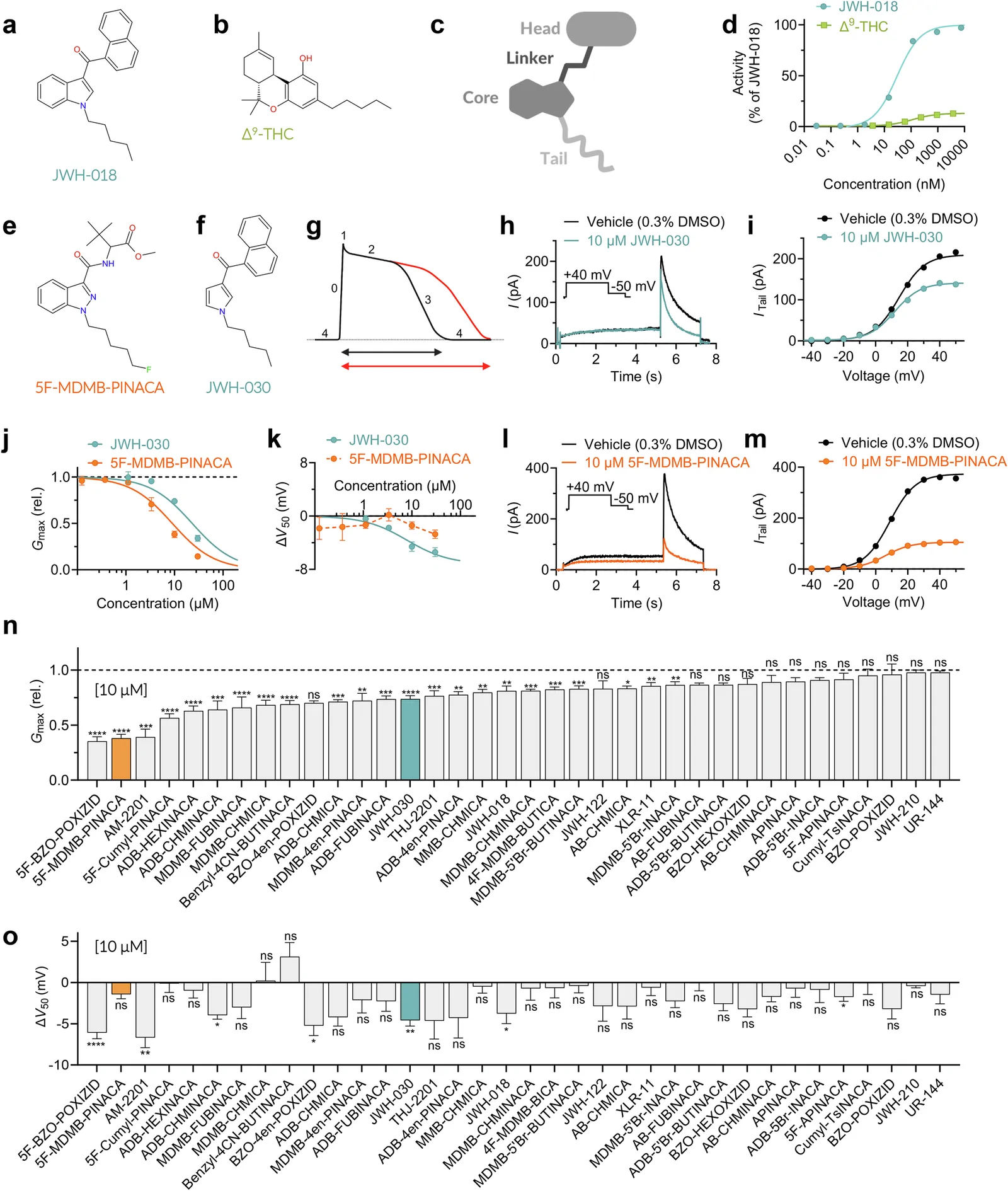
The majority of tested SCRAs inhibit hERG. Chemical structure of the SCRAs JWH-018 (**a**) and Δ^9^-THC (**b**). **c** Schematic illustration of the division of a generic SCRA into four key building blocks: the head, linker, core, and tail. **d** CB_1_ receptor activation of JWH-018 (*n* = 3) and Δ^9^-THC (*n* = 3), where data are normalized to the maximal response of JWH-018 (used as reference), arbitrarily set at 100%. Chemical structures of the SCRAs 5F-MDMB-PINACA (**e**) and JWH-030 (**f**). **g** Schematic illustration of an action potential from a ventricular cardiomyocyte, with its five phases (0-4) indicated, where phase 3 represents the repolarization phase. The solid black line represents an action potential under normal conditions, the solid red line represents action potential prolongation due to compromised repolarization (e.g., reduced *I*_Kr_ or *I*_Ks_). Representative effect of JWH-030 on hERG using the voltage protocol shown as inset (**h**), with corresponding *G*(*V*) curve (**i**). Concentration-response relationship for *G*_max_ (rel) (**j**) or *V*_50_ (**k**) effect of JWH-030 and 5F-MDMB-PINACA. *n* = 6-8 for JWH-030; *n* = 4-17 for 5F-MDMB-PINACA. **l, m** Same as in (**h, i**) but for 5F-MDMB-PINACA. Average effect of 10 µM of the indicated SCRAs on *G*_max_ (**n**) and *V*_50_ of hERG (**o**). *n* = 3-13. Statistical comparisons were performed versus time-matched vehicle controls. For comparisons involving a single compound versus vehicle, Welch’s *t*-test was used. For comparisons involving multiple compounds sharing the same vehicle control, one-way ANOVA (no matching) followed by Dunnett’s multiple comparisons test was applied. Statistics denote adjusted *P*-values for comparisons to vehicle: *P* < 0.05 (*), *P* < 0.01 (**), *P* < 0.001 (***), *P* < 0.0001 (****). All average data are shown as mean ± SEM. Small error bars are covered by symbols. Best-fit parameters are provided in SI Table II; quality control criteria for fits are summarized in SI Table III.

Synthetic cannabinoid receptor agonists (SCRAs, exemplified by JWH-018 in Fig. 1a), commonly known as “spice” or “K2”, are a large class of new psychoactive substances (NPS) designed to mimic the effects of Δ^9^-tetrahydrocannabinol (Δ^9^-THC) (Fig. 1b), the main psychoactive component of cannabis [1]. The SCRA available on the recreational drug market is constantly changing with new compounds emerging each year to circumvent national and international legislation, posing international threats and challenges to the communities. For newly emerging SCRAs, chemical modifications may be seen at the core, tail, head (also known as linked group), or linker of the SCRA molecule (Fig. 1c). As of 2025, 277 different SCRAs have been reported to the European Union early warning system, and it is the largest group of NPS being monitored by the European Union Drug Agency (EUDA) [2].

SCRAs have been estimated to be used by 0.2%–4% of the population [3], with much higher use reported in prisons [4], rough-sleeping populations [5], and populations with regular drug testing, including the military [6] and people on probation/parole [7]. SCRAs are often smoked as infused herbal material or vaped as infused e-cigarette oils [8]. There have also been many reports of the addition of SCRAs to cannabis products on the market [9, 10] and SCRAs found in drug samples sold as opioids or benzodiazepines [11], meaning people who use SCRAs are often unaware of the actual SCRAs they are using or may not even know they are using SCRAs. The high variability of the market and lack of pharmacological data of newly emerging drugs significantly increase the risk of harm.

SCRAs are typically full agonists of the cannabinoid 1 (CB_1_) receptor, the receptor responsible for the psychoactivity of cannabis, whereas Δ^9^-THC is only a partial agonist [1]. SCRAs are often significantly more potent on the CB_1_ receptor than Δ^9^-THC (exemplified by JWH-018 in Fig. 1d) and have many adverse effects reminiscent of psychostimulants rather than cannabis, including psychosis, seizures, and cardiac arrhythmia [12]. Cardiovascular-related complications of SCRAs are the most common symptoms presented at accident and emergency departments [13, 14] and common causes of death [15–17]. For instance, 5F-MDMB-PINACA (Fig. 1e, also known as 5F-ADB), has been implicated in several fatal intoxications [18–20] and linked to cardiac symptoms [21]. Thus, there is a substantial body of evidence linking SCRA intoxication to potentially fatal cardiac arrhythmias [22], with QT prolongation on the electrocardiogram (ECG) reported in several studies [e.g., 23–25].

Despite frequent reports associating SCRA intoxication with arrhythmias, little is known about how SCRAs cause these adverse effects on a mechanistic level [22]. As cardiac arrhythmias are electrophysiological disorders, cardiac ion channels may be implicated. As the first example supporting this, an electrophysiological study reported that JWH-030 (Fig. 1f) inhibits the voltage-gated potassium channel hERG (K_V_11.1) and prolongs the QT interval in rats [26]. Moreover, a recent integrative computational and electrophysiological study shows that 5F-APINACA (also called 5F-AKB48) and ADB-FUBIATA inhibit hERG and find a side pocket lateral to the central cavity for these two SCRAs [27]. Another study used machine learning models to propose hERG binding of several SCRAs, though no electrophysiological experiments were conducted to confirm the predictions [28]. These three studies provide the first indication of the interaction between SCRAs and hERG, highlighting the importance of more extensive studies.

The hERG channel, together with the K_V_7.1/KCNE1 channel, is responsible for phase 3 repolarization of the action potential in ventricular cardiomyocytes: hERG carries the rapidly activating delayed rectifier current *I*_Kr_, whereas K_V_7.1/KCNE1 carries the slowly activating delayed rectifier current *I*_Ks_ (Fig. 1g). Impaired function of hERG or K_V_7.1/KCNE1 can lead to a prolonged action potential duration (indicated in red in Fig. 1g), early afterdepolarizations and arrhythmic beats [29, 30]. This is observed in ventricular arrhythmias such as Torsades de Pointes, a life-threatening polymorphic tachycardia that is a consequence of both drug-induced Long QT Syndrome (LQTS) following hERG channel inhibition and as congenital LQTS caused by deleterious mutations in the genes encoding hERG and K_V_7.1/KCNE1 [29, 30]. However, whether SCRAs other than JWH-030, 5F-APINACA, and ADB-FUBIATA inhibit hERG remains unclear. In addition, putative SCRA effects on K_V_7.1/KCNE1 remain unknown. Thus, our study aims to address this knowledge gap by assessing the effects of a broad set of SCRAs on hERG and K_V_7.1/KCNE1 channels.

## Results

### SCRAs inhibit the hERG channel by reducing channel conductance

To assess the effect of a large set of SCRAs on hERG (see SI Table I and SI Fig. S1 for a list of the 36 included SCRAs and corresponding structures), we used the automated planar patch-clamp (APC) technique on mammalian Chinese hamster ovary (CHO) cells stably expressing human hERG. All 36 SCRAs were evaluated in concentration-response experiments using concentrations ranging from 1.11 to 30 µM (also lower concentrations were tested for potent compounds). We employed a voltage-ladder protocol to obtain information on SCRA effects on the channel’s voltage dependence of activation (*V*_50_) and maximal conductance (*G*_max_) (both determined from Boltzmann fits of the instantaneous tail currents; see Methods for details). SCRA efficacy (e.g., maximal reduction in *G*_max_) and potency (SCRA concentration required to produce 50% of the compound’s maximal effect; IC_50_) were determined (see Methods for details). In line with previous reports, JWH-030 inhibited hERG (Fig. 1h, i), seen in our assay as a concentration-dependent decrease in *G*_max_ (Fig. 1j, IC_50_ = 23.1 µM, 95% CI = 16.1–29.7) and a small effect on *V*_50_ (Fig. 1k). Among the tested compounds, 5F-MDMB-PINACA was one of the more potent inhibitors, reducing *G*_max_ ﻿of hERG at lower concentrations than JWH-030 (Fig. 1j–m, IC_50_ = 9.0 µM, 95% CI = 4.8-9.9).

Most SCRAs displayed inhibitory effects on hERG, primarily in the form of decreased *G*_max_ (see SI Fig. S2 for concentration-response data and SI Table II for efficacy and potency). For several compounds, the concentration-response curves did not saturate at the highest concentration tested (SI Fig. S2), resulting in suboptimal curve fitting with unreliable efficacy and potency estimates (SI Table II–III). We therefore compared the effects of the 36 SCRAs at 10 µM, a concentration that provided a consistent and representative estimate of the overall inhibitory effect on hERG. At 10 µM, 23 of the SCRAs significantly reduced *G*_max_ (Fig. 1n). Corresponding manual patch-clamp experiments yielded comparable effects for three tested SCRAs (SI Fig. S3a). In contrast, at 10 µM only 7 of the tested SCRAs induced a significant shift in *V*_50_ (Fig. 1o). Also, the magnitude of these *V*_50_ shifts was modest, with all Δ*V*_50_ confined within a narrow range of ± 7 mV. Hence, for the rest of the study, we will focus on the *G*_max_ response of hERG to the SCRAs.

### SCRA building block chemistry impacts SCRA inhibition of hERG

**Fig. 2:**
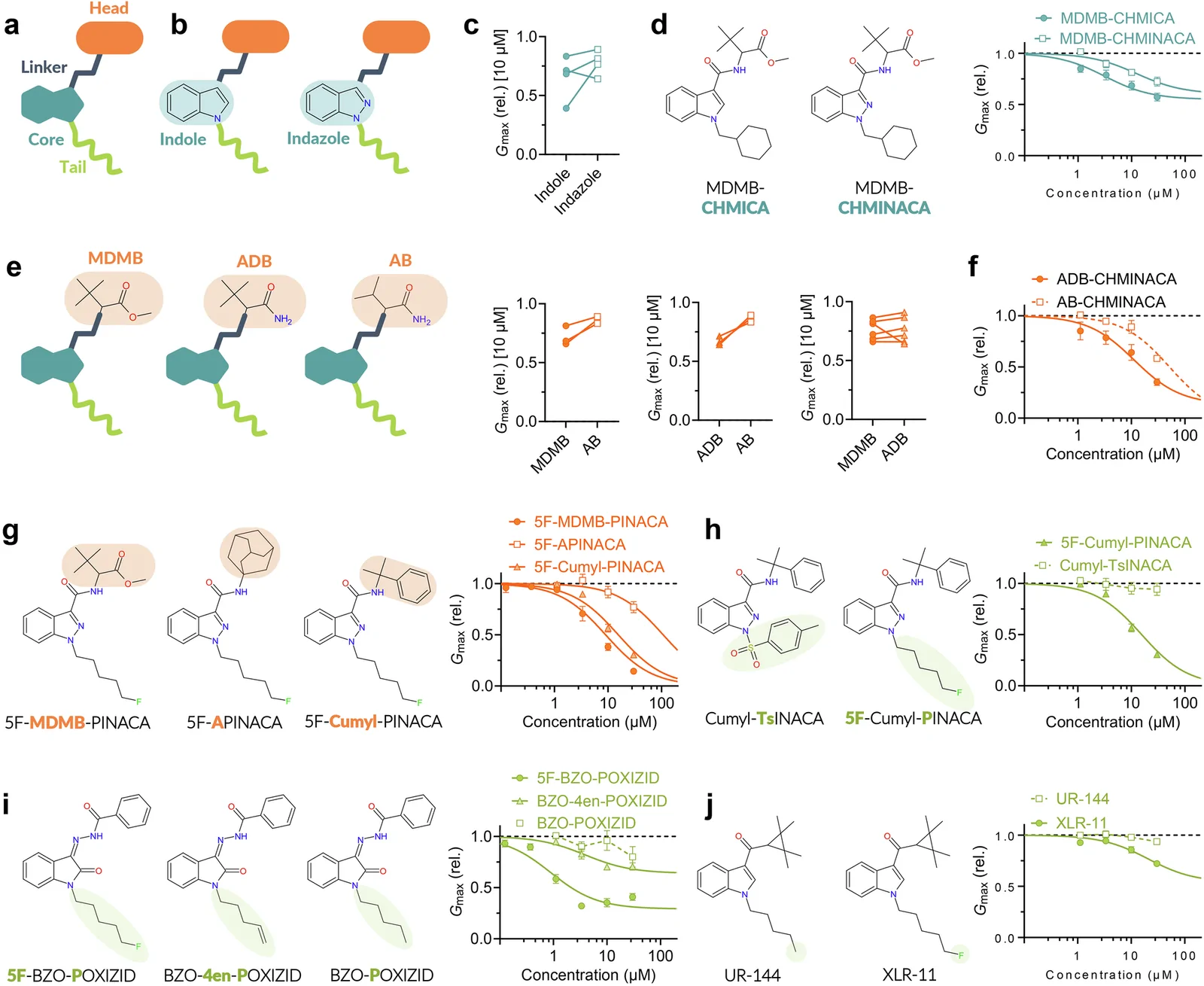
Chemical modifications to the SCRA building blocks impact hERG effects. **a** Schematic illustration of the four key building blocks of SCRAs colour coded as in subsequent illustrations. **b** Schematic illustration of SCRAs with indole and indazole cores, together with comparison of effect at 10 µM. *n* = 4–9. **c,** **d** Example of impact of core moiety on hERG inhibition, illustrated by MDMB-CHMICA and MDMB-CHMINACA. *n* = 4–5. Additional examples are shown in Fig. S4. **e** Schematic illustration of SCRAs with indicated heads, together with a comparison of the effect at 10 µM. *n* = 4–7. **f** Example of the impact of the head moiety showcased by ADB-CHMINACA and AB-CHMINACA for hERG inhibition. *n* = 4. Additional examples are shown in Fig. S4. **g** Molecular structures of three SCRAs with 5F-PINACA scaffolds and variable head groups, and concentration-response relationships for indicated SCRAs on hERG inhibition. *n* = 4–17. **h** Molecular structures of two SCRAs with Cumyl-INACA scaffolds and variable tails, and concentration-response relationships for indicated SCRAs on hERG inhibition. *n* = 4–7. **i** Molecular structures of three SCRAs with BZO-OXIZID scaffolds and variable tails, and concentration-response relationships for indicated SCRAs on hERG inhibition. *n* = 5–12. **j** Molecular structures of two SCRAs with variable tails, and concentration-response relationships for indicated SCRAs on hERG inhibition. *n* = 4–10. All average data are shown as mean ± SEM. Small error bars are covered by symbols. Best-fit parameters are provided in SI Table II; quality control criteria for fits are summarized in SI Table III.

We noted several interesting examples of how chemical modifications to the building blocks (Fig. 2a) of SCRAs influenced the hERG inhibitory effects on *G*_max_. However, detailed structure-activity relationship (SAR) analysis for efficacy and potency was not possible because of the unreliable concentration-response fits of *G*_max_ reduction in hERG for several SCRAs (SI Table II–III). For the core, we noted two examples for which SCRAs with an indole core showed more prominent effects on hERG than the corresponding SCRAs with an indazole core (Fig. 2b, c; SI Fig. S4). This is exemplified by MDMB-CHMICA vs MDMB-CHMINACA in Fig. 2d. However, two other examples showed overall comparable hERG effects for SCRAs with indole and indazole cores (Fig. 2c; SI Fig. S4), suggesting no consistent pattern across the cores. Of the different amino acid-derived head moieties (Fig. 2e), SCRAs with tert-leucine methyl esters (MDMB) and tert-leucinamides (ADB) showed a trend towards more prominent hERG effects compared to valinamide (AB) SCRAs (Fig. 2e, f; SI Fig. S4). Moreover, the 5F-PINACA scaffold with either a branched MDMB or aromatic cumyl head group induced concentration-dependent inhibitory effects on *G*_max_, whereas the bulky adamantyl in 5F-APINACA impaired the inhibitory effect (Fig. 2g). As another example, a bulky tosyl group at the tail of the Cumyl-INACA scaffold caused Cumyl-TsINACA to be a non-inhibitor (Fig. 2h). Furthermore, addition of a terminal fluorine (5F-pentyl in 5F-BZO-POXIZID) or alkene (pent-4-enyl in BZO-4en-POXIZID) on the pentyl tail of the BZO-POXIZID scaffold produced concentration-dependent inhibitory effects on *G*_max_, whereas a simple pentyl tail (in BZO-POXIZID) rendered this SCRA a non-inhibitor (Fig. 2i). As another example of tail chemistry, terminal fluorination of the pentyl tail on the UR-144 scaffold (i.e., XLR-11) resulted in concentration-dependent inhibitory effects on *G*_max_, whereas its non-fluorinated counterpart (i.e., UR-144) was a non-inhibitor (Fig. 2j). Similarly, 5F-APINACA showed inhibitory effects on *G*_max_, whereas APINACA was a non-inhibitor (SI Table II). Altogether, the screening of 36 SCRAs on the hERG channel demonstrates that specific structural elements markedly influence hERG inhibition.

### SCRAs may use both a promiscuous central pore cavity site and lateral side pocket sites in hERG

**Fig. 3:**
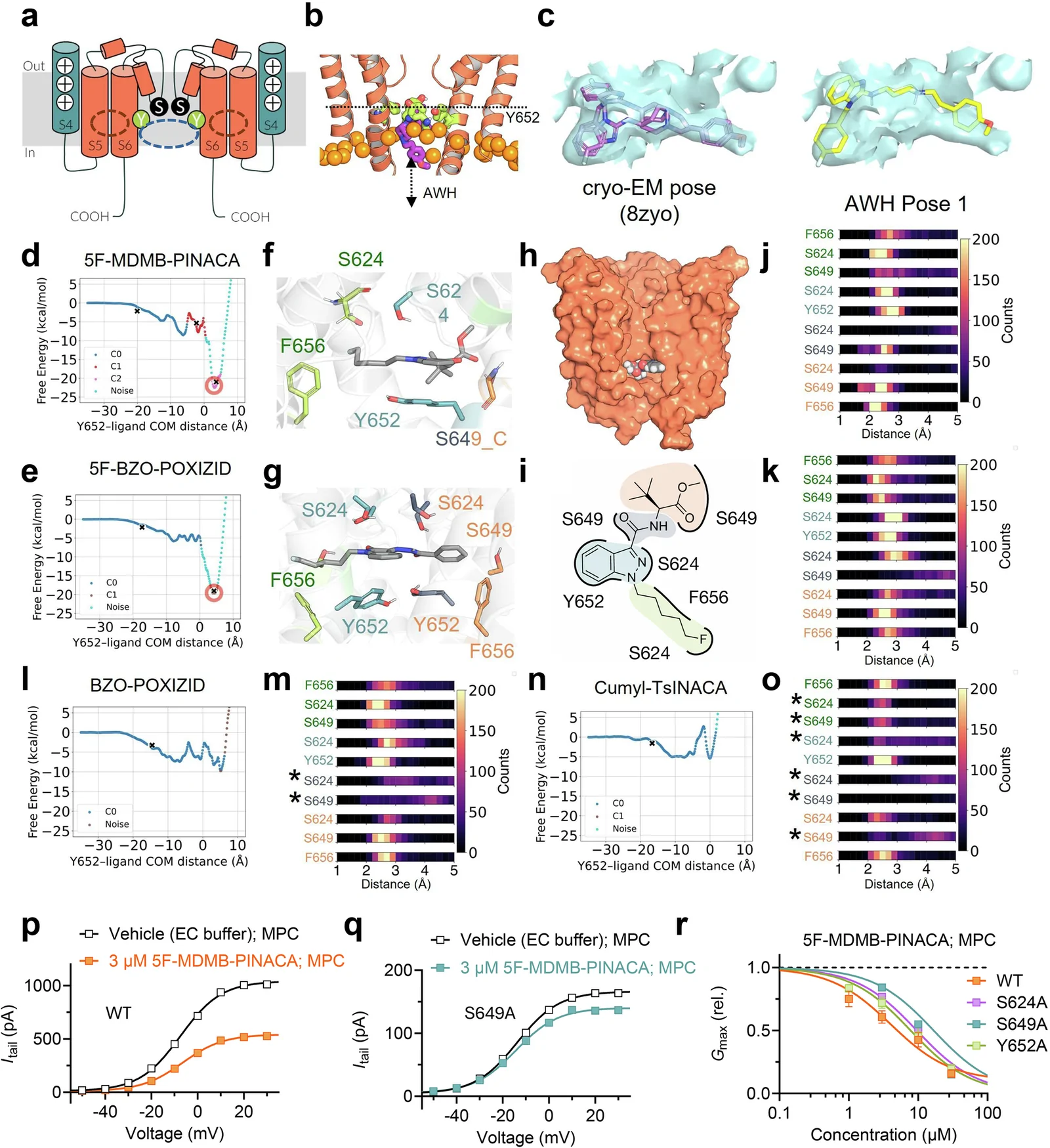
Accelerated weighted histogram simulations provide insight into SCRA binding poses. **a** Simplified schematic illustration of hERG (side view) indicating the central ion-conducting pathway delineated by the selectivity filter, S624 (denoted S), and Y652 (denoted Y) in the central cavity. Pore domain regions are in orange and the voltage-sensor S4 in teal. The central cavity site is indicated in dashed blue and the lateral side pocket site in dashed dark orange. **b** Structural representation of the path for astemizole (purple) to sample along the z-axis of hERG (orange) with respect to Y652 (green). **c** Electron density map obtained from EMD-60574 (cyan) overlayed on the cryo-EM structure of astemizole (purple), compared to the pose obtained from the minima by AWH (yellow). **d** Free energy landscape of the hERG inhibitor 5F-MDMB-PINACA sampling along the z-axis with respect to Y652, starting from the pose based on astemizole. A red circle and a cross mark the minima of the landscape. Different colours indicate different clusters extracted from DBSCAN with the epsilon = 0.2 with at least 10 frames in the cluster. **e) **Same as in **d** but for 5F-BZO-POXIZID. Snapshots of 5F-MDMB-PINACA (**f**) and 5F-BZO-POXIZID (**g**) from the cluster centre of the free energy minima. The C-atoms of the SCRA are coloured gray. Residues interacting with the SCRAs are coloured according to which building block they interact with, with the head group in orange, the linker in black, the core in blue and the tail in green (same colour coding as in Fig. 2). **h** A zoom-out picture of the SCRA binding site on the pore domain of hERG (shown as an orange surface). 5F-MDMB-PINACA is shown as a black sphere with CPK colouring for the elements. **i** Schematic representation of SCRAs in the binding pocket (here illustrated for 5F-MDMB-PINACA), forming interactions with residues on hERG subunits. Distribution of minimum distance analysis from 1000 structures at the minima performed between each residue in four hERG subunits and the four building blocks of the SCRAs for 5F-MDMB-PINACA (**j**) and 5F-BZO-POXIZID (**k**). Residue labels on the left side are colour-coded according to the building block they contact (same colours as in **f, g**). The number of contacts each interaction is shown on the colour bar on the right side. The bin size of the histogram is 20. **l, m** Same as in (**d, j**) but for the non-inhibitor BZO-POXIZID. **n, o** Same as in panels **d** and **j** but for the non-inhibitor Cumyl-TsINACA. The asterisks mark residues with no contact. Representative effect of 3 µM 5F-MDMB-PINACA on hERG wild type (WT) (**p**, stable cell line) or hERG S649A (**q**, transient cell line) recorded using MPC. Current traces and representative effects on S624A and Y652A are shown in Fig. S2b, e. **r** Concentration-response relationship for the *G*_max_ (rel) effect of 5F-MDMB-PINACA on hERG WT (orange), S624A (purple), S649A (teal), and Y652A (green), collected using MPC. Best fits for *G*_max_ (rel) yielded IC_50_ values of 4.0 µM (95% CI = 2.2–6.8 µM; *n* = 14–24) for WT, 9.6 µM (95% CI = 7.6–11.2 µM; *n* = 4–5) for S624A, 16.7 µM (95% CI = 4.7–19.0 µM; *n* = 4–6) for S649A and 7.8 µM (95% CI = 4.4–10.0 µM; *n* = 9–14) for Y652A. The IC_50_ values were not significantly different (*P* > 0.05) using the Extra sum-of-squares *F*-test.

hERG has a known, promiscuous canonical binding site in the central cavity of the pore domain that is used by many inhibitors and blockers [31, 32], including the histamine receptor antagonist and hERG blocker astemizole [32–34] (see also SI Fig. S5 for inhibitory effect of astemizole in our hERG assay). This central cavity site is just below the selectivity filter and flanked by aromatic residues, like Y652 (schematic illustration in Fig. 3a). Moreover, a recent study revealed a noncanonical inhibitory site in hERG used by 5F-APINACA [27], here referred to as the side pocket site (schematically illustrated in Fig. 3a). This site is lateral to the central cavity site (on the other side of Y652 compared to the central cavity site) and flanked by residues like F557 and M651 [27]. To assess whether SCRAs may interact with hERG in the central cavity and side pocket sites and predict the mode of interaction, we started by docking astemizole (as a positive control for the central cavity site) and all 36 SCRAs to the recently solved structures with astemizole density (PDB entry: 8ZYO, astemizole structure being built in; PDB entry: 7CN1, astemizole structure not being built in). The docking was centered at Y652, which is close to both the central cavity site and the side pocket site (SI Fig. S6a). Using the 7CN1 structure, the docked pose of astemizole and the SCRAs were residing on the side pocket (SI Fig. S6b), similar to what was seen for 5F-APINACA in the recent study [27]. However, the docking score was relatively low, suggesting low binding affinity at the side pocket (SI Fig. S6b). Interestingly, when docked to the 8ZYO structure, all SCRAs’ primary binding sites were located in the central cavity site (SI Fig. S6c). By instead centering the docking at F557 in the 8ZYO structure, SCRAs also found the side pocket site, although with lower affinity than in the central cavity site (SI Fig. S6d). This is interesting, given the relatively small structural differences between 7CN1 and 8ZYO in the region of interest, with the main difference being the rotamer of Y652. The redocking of astemizole to the binding pocket in 8ZYO showed a near match to the experimental structure (SI Fig. S6c). Together, these highlight the possibility that both binding sites can be used alternatively for hERG inhibition by SCRA. However, the binding free energy from docking (SI Fig. S6) showed no correlation with the experimental data, with SCRAs inducing no-to-prominent hERG inhibition in experiments despite comparable binding free energy from docking.

Given the discrepancy in the binding pose arising from the side-chain rotamers and a slight difference in protein conformation, we developed a novel computational pipeline using accelerated weighted histogram (AWH) simulations, with astemizole as a positive control (SI Fig. S7). This AWH simulation used 7CN1 as the starting protein structure, with astemizole randomly placed in the central cavity of the pore. The work from the pipeline resulted in the binding poses of astemizole matching the recently solved cryo-EM density on the 8ZYO structure [35] with astemizole binding perpendicular to the ion conducting pathway in the central cavity site, about 5 Å above Y652 (Fig. 3b, c, SI Fig. S7). From this site, astemizole blocks hERG by preventing potassium ion permeation [35]. This highlighted the advantage of AWH, as the success of the method in predicting the binding site was independent of the side chain rotamers of the initial structure. We followed the same workflow for four SCRAs, two of which were inhibitors in our hERG assay by reducing *G*_max_ (5F-MDMB-PINACA and 5F-BZO-POXIZID) and two of which failed to inhibit the channel (BZO-POXIZID and Cumyl-TsINACA), to gain insight into the inhibitory effect at the central cavity site. Like astemizole, the simulations with the inhibitors 5F-MDMB-PINACA and 5F-BZO-POXIZID found an energy minimum at 5 Å above Y652 (Fig. 3d, e). Given the time constraint in the AWH simulations, we could not achieve a converged free energy landscape (SI Fig. S8). However, the poses clustered from the energy minima showed the SCRAs in the cavity in a planar orientation perpendicular to the ion conduction pathway, sandwiched between Y652 and the selectivity filter (Fig. 3f, g for 5F-MDMB-PINACA and 5F-BZO-POXIZID, respectively, and Fig. 3h for a zoomed-out representation of 5F-MDMB-PINACA). This perpendicular conformation is very similar to that of astemizole, suggesting prevention of potassium ion permeation. We then took 1000 snapshots from the minima of the cluster to analyse key contacting residues, leading to: (i) the identification of Y652 interacting with the SCRA core by pi-pi stacking; (ii) S624 and S649 interacting with either the head or the tail of the SCRA, providing hydrogen bonds; and (iii) F656 interacting with the alkyl tail by hydrophobic interaction, or defining the size of the head of the SCRAs that can be accommodated in the binding pocket (Fig. 3i). All snapshots from the minima showed the distribution being mostly unimodal, suggesting a very stable binding pose (Fig. 3j, k). Also, for the non-inhibitor BZO-POXIZID, we found an energy minimum at 5 Å above Y652 (Fig. 3l). However, while we identified a hydrogen bond between the hydroxyl group on S624 and/or S649 and the fluorine of the 5F-pentyl tails of 5F-MDMB-PINACA and 5F-BZO-POXIZID, these were absent for BZO-POXIZID and may explain why it is a non-inhibitor. Hence, although S624 and/or S649 remain in close proximity with the tail in BZO-POXIZID (Fig. 3m), they are unable to form any H-bonding, which is expected to result in a weaker affinity to the channel. Lastly, we sought insight into the structural basis of the non-inhibitor Cumyl-TsINACA. The free energy landscape from the simulations indicates that Cumyl-TsINACA did not show a minimum at 5 Å position (Fig. 3n). This placed both S624 and S649 out of the head binding pocket, misplaced the linker region, and disrupted the interaction with the tail (Fig. 3o, with main lost interactions highlighted by *). Together, these suggested how Cumyl-TsINACA fails to prevent potassium ion permeation.

Based on the above-described SCRA poses in the central cavity and the experimental data, we hypothesize that a larger head group (e.g., adamantyl) or tail group (e.g., tosyl) prevents the SCRA from binding properly, resulting in a shift in the position of the minima in the free energy landscape. Also, it may be that either the head group (MDMB or Cumyl) or the fluorinated tail must be able to form hydrogen bonds with S624 or S649, such that SCRAs with no tail (like MDMB-5’Br-INACA or ADB-5’Br-INACA) will result in a weaker interaction with the binding pocket. Given the limitation on the convergence of the free energy in the simulations, it is challenging to determine how the small differences between the indole and indazole cores impact the pi-stacking interaction with Y652. However, indazole has been shown to be less electron-rich than indole due to the additional p-orbital on the N atom [36]. This may weaken the pi-stacking interactions with Y652 for some SCRAs.

Mutating S624, S649, and Y652 to alanine may impact the interaction between SCRAs and these residues in the central cavity. We compared the 5F-MDMB-PINACA effect on hERG wild type and the S624A, S649A and Y652A mutants. 5F-MDMB-PINACA still exerted prominent inhibitory effects on *G*_max_ on these mutants (Fig. 3p–r; SI Fig. S3b, e), with no differences in potency. That 5F-MDMB-PINACA still exerts a clear inhibitory effect on these mutated channels could indicate preserved or compensatory interactions with other residues in this site, or primary or compensatory inhibiting effects arising from the side pocket site.

### Comparing SARs for SCRA effects on hERG and the CB_1_ receptor reveals similarities and differences

**Fig. 4:**
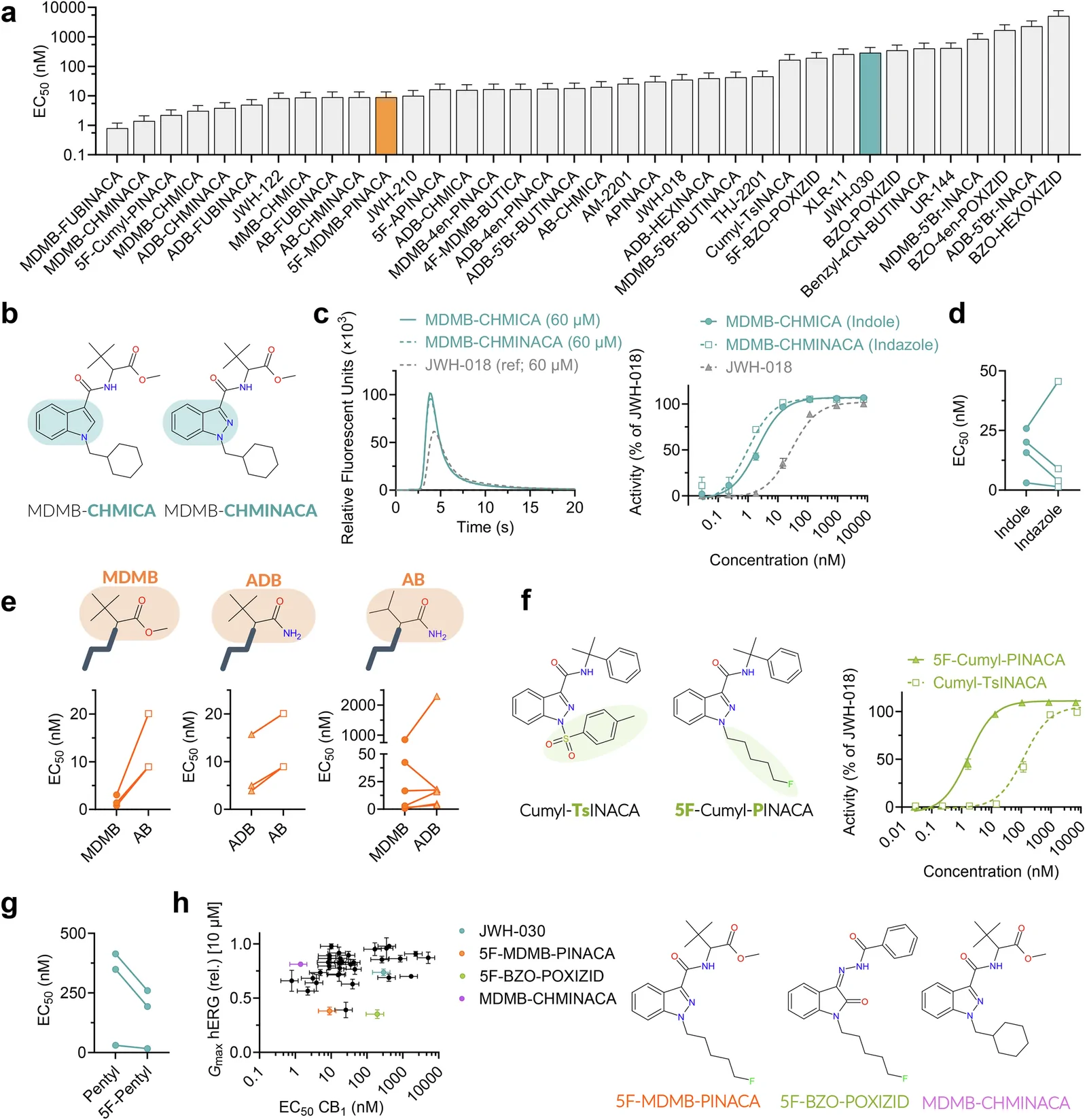
Comparison of SCRA features important for CB_1_ and hERG effects reveal similarities and differences. **a** Potency of the indicated SCRAs on CB_1_ (see methods for details). *n* ≥ 3 (except from JWH-030 where *n* = 2). **(b-c)** Example of impact of core moiety for CB_1_ activation showcased by MDMB-CHMICA and MDMB-CHMINACA (*n* = 3; structures in (**b**) and CB_1_ activating effects in (**c**). **d** Outcome of SAR analysis (paired *t*-test, α = 0.05) of SCRAs with indole and indazole cores (*n* = 4) for CB_1_ potency. **e** Schematic illustration of SCRAs with indicated heads, together with the outcome of SAR analysis (paired *t*-test, α = 0.05; *n* = 3, 3, and 5, respectively) for CB_1_ potency. **f** Molecular structures of two SCRAs with Cumyl-INACA scaffolds and variable tails, and concentration-response relationships for indicated SCRAs on CB_1_ activity (*n* = 4 for 5F-Cumyl-PINACA and *n* = 5 for Cumyl-TsINACA). **g** Outcome of SAR analysis of SCRAs with non-fluorinated compared with fluorinated pentyl tails for CB_1_ potency. **h** Comparison of *G*_max_ response of hERG induced by SCRAs at 10 µM (y-axis) and CB_1_ potency (x-axis). Specific SCRAs are highlighted as indicated in the figure. All average data are shown as mean ± SEM. Small error bars are covered by symbols. CB_1_ data are from a minimum of three independent experiments (*n* ≥ 3), run in triplicate (except for JWH-030, for which *n* = 2).* n*for hERG = 3-13. Best-fit parameters are provided in SI Table II.

To explore the relationship between the potential cardiotoxic effects of SCRAs via hERG and their psychoactive effects mediated by the CB_1_ receptor, we assessed the activity of the 36 SCRAs on CB_1_ stably expressed in CHO-K1 cells using an AequoScreen^®^ intracellular calcium release assay (see Methods for details; SI Fig. S9a for an exemplary example of JWH-018). The compounds showed a wide range of potencies on CB_1_, with EC_50_ values spanning from sub-nanomolar (e.g., MDMB-FUBINACA) to low micromolar levels (e.g., BZO-HEXOXIZID) (Fig. 4a; SI Table II).

In contrast to the results on hERG, MDMB-CHMICA and MDMB-CHMINACA showed similar effects on CB_1_ (Fig. 4b, c; SI Table IV). No consistent pattern in CB_1_ potency was observed between compounds with indole or indazole cores (Fig. 4d). For the head moiety, a similar overall pattern was seen for CB_1_ receptor activity as for hERG, where tert-leucine methyl esters (MDMB) and tert-leucinamides (ADB) were consistently more potent than the corresponding valinamide (AB) head moiety (Fig. 4e; SI Table V). Moreover, an adamantyl group (5F-APINACA) instead of an MDMB or cumyl group reduced the hERG inhibitory *G*_max_ effect and also reduced the CB_1_ potency (SI Fig. S9b).

Similar to the results on hERG, replacing the fluorinated pentyl tail of 5F-Cumyl-PINACA with the tosyl tail of Cumyl-TsINACA reduced the effect at the CB_1_ receptor, shifting the EC_50_ from 2.2 nM to 166 nM (Fig. 4f). For hERG, the importance of fluorination was further supported by the increased effect of 5F-BZO-POXIZID compared to BZO-POXIZID, and similarly for UR-144 versus XLR-11. At the CB_1_ receptor, a similar importance of tail fluorination was seen, as fluorinated SCRAs tended to show higher potency compared to their non-fluorinated counterparts (Fig. 4a, g; SI Table VI). Taken together, certain structural features, particularly in the head and tail moieties, show similarities in how they contribute to effects in hERG and CB_1_. However, the overall ability of SCRAs to affect hERG and CB_1_ shows little apparent association (Fig. 4h), with some SCRAs affecting both (e.g., 5F-MDMB-PINACA in orange), some having effects primarily on hERG (e.g., 5F-BZO-POXIZID in green) or primarily on CB_1_ (e.g., MDMB-CHMINACA in purple), reflecting distinct SARs.

### Several SCRAs show dual inhibitory effects by targeting both hERG and K_V_7.1/KCNE1

**Fig. 5:**
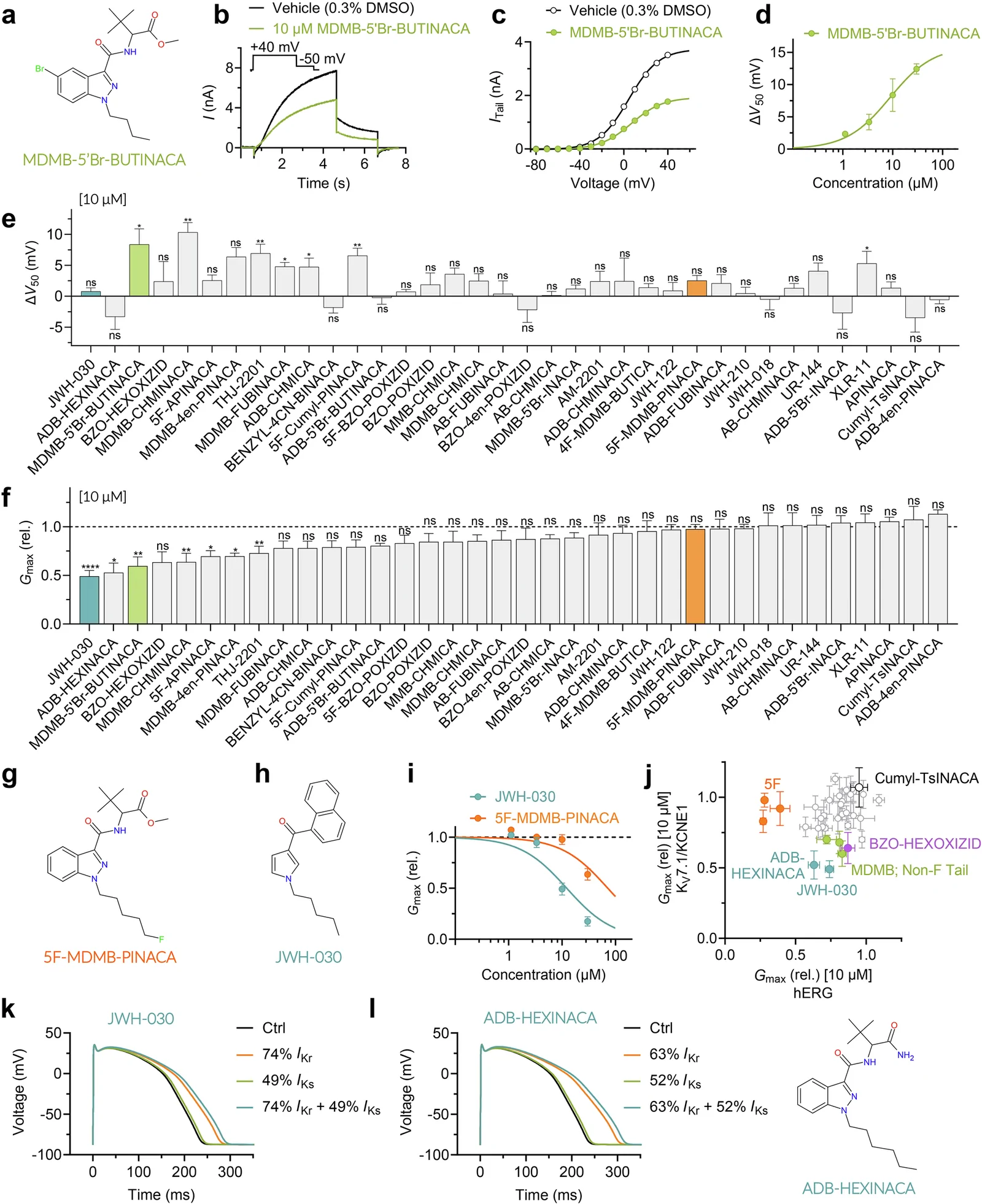
Some tested SCRAs also inhibit K_V_7.1/KCNE1. **a** Chemical structure of MDMB-5’Br-BUTINACA. In **b**, representative effect of 10 µM MDMB-5’Br-BUTINACA on K_V_7.1/KCNE1 using the voltage protocol shown as inset with corresponding *G*(*V*) curve (**c**). **d** Concentration-response relationship for *V*_50_ effect of MDMB-5’Br-BUTINACA on K_V_7.1/KCNE1. *n* = 4–5. Average effect of 10 µM of the indicated SCRAs on *V*_50_ (**e**) and *G*_max_ (**f**) of K_V_7.1/KCNE1. *n* = 4–9. Statistical comparisons were performed versus time-matched vehicle controls. For comparisons involving a single compound versus vehicle, Welch’s *t*-test was used. For comparisons involving multiple compounds sharing the same vehicle control, one-way ANOVA (no matching) followed by Dunnett’s multiple comparisons test was applied. Homogeneity of variance was assessed using the Brown–Forsythe test, and when significant heteroscedasticity was detected (*P* < 0.05), Welch’s ANOVA followed by Dunnett’s T3 multiple comparisons test was used instead. Statistics denote adjusted *P*-values for comparisons to vehicle: *P* < 0.05 (*), *P* < 0.01 (**), *P* < 0.001 (***), *P* < 0.0001 (****). Chemical structure of 5F-MDMB-PINACA (**g**) and JWH-030 (**h**) with concentration-response relationship for their rel. *G*_max_ effects on K_V_7.1/KCNE1 (**i**). *n* = 6. **j** Comparison of *G*_max_ response of K_V_7.1/KCNE1 (y-axis) and hERG (x-axis) induced by SCRAs at 10 µM. Specific SCRAs are highlighted as indicated in the figure. Three SCRAs with 5F-pentyl tails (5F-MDMB-PINACA, 5F-BZO-POXIZID, AM-2201) are indicated in orange. Three SCRAs with MDMB heads and non-fluorinated tails (MDMB-5’Br-BUTINACA, MDMB-4en-PINACA, MDMB-CHMINACA) are indicated in green. Simulated ventricular action potential in wild type cardiomyocytes under control conditions (black) and with the experimentally observed effects of 10 µM JWH-030 (**k**) or ADB-HEXINACA (**l**) on hERG alone (denoted *I*_Kr_, with numbers indicating remaining *I*_Kr_), K_V_7.1/KCNE1 alone (denoted *I*_Ks_, with numbers indicating remaining *I*_Ks_), or both (denoted *I*_Kr_ + *I*_Ks_). Control (Ctrl) denotes unaffected *I*_Kr_ and *I*_Ks_ (i.e., 100 %). All average data are shown as mean ± SEM. Small error bars are covered by symbols. Concentration-response relationships are provided in SI Fig. S10 and S11. Best-fit parameters are provided in SI Table II.

Both hERG and K_V_7.1/KCNE1 contribute to cardiomyocyte repolarization (Fig. 1g). Inhibition of K_V_7.1/KCNE1 alone can be proarrhythmic [37]. Moreover, under conditions with impaired hERG function, upregulation of K_V_7.1/KCNE1 has been proposed to partially compensate for the loss of hERG [38, 39]. Hence, drugs that inhibit both hERG and K_V_7.1/KCNE1 may have compounded proarrhythmic effects [40]. To assess if SCRAs also affect K_V_7.1/KCNE1, we used mammalian CHO cells stably expressing human K_V_7.1/KCNE1. At 10 µM, several SCRAs displayed inhibitory effects on K_V_7.1/KCNE1, seen as reduced *G*_max_ and/or shifted *V*_50_ (SI Fig. S10, 11). A notable example was MDMB-5’Br-BUTINACA (Fig. 5a), which both reduced *G*_max_ and shifted *V*_50_ towards more positive voltages (Fig. 5b–d). At 10 µM, 7 of the SCRAs caused a significant positive shift in *V*_50_ (Fig. 5e), with the largest shift induced by MDMB-CHMINACA (Δ*V*_50_ = +10.3 ± 1.6 mV). At 10 µM, 7 of the SCRAs significantly reduced *G*_max_ (Fig. 5f). Several SCRAs produced significant effects on both *G*_max_ and *V*_50_ at 10 µM; however, only 5F-Cumyl-PINACA and THJ-2201 exhibited well-defined concentration–response relationships for both effects (SI Fig. S10, 11; SI Table II). In contrast to hERG, 5F-MDMB-PINACA had a small effect on K_V_7.1/KCNE1, while JWH-030 induced the largest reduction in *G*_max_ (Fig. 5e–i; SI Table II). Hence, several SCRAs inhibited K_V_7.1/KCNE1; however, they were fewer and induced less prominent reduction in *G*_max_ compared to what was observed for hERG.

Given the limited number of SCRAs with effects on K_V_7.1/KCNE1, no SAR analysis could be performed. However, we note several interesting clusters of compounds, exemplified for 10 µM on *G*_max_ in Fig. 5j. At 10 µM, some SCRAs had no effect on either of the two channels (e.g., Cumyl-TsINACA in black), whereas some had effects only on hERG (e.g., 5F-MDMB-PINACA, 5F-BZO-POXIZID, and AM-2201 in orange) or only on K_V_7.1/KCNE1 (e.g., 5F-APINACA in purple). Yet other SCRAs had inhibitory effects on both hERG and K_V_7.1/KCNE1 (e.g., JWH-030 and ADB-HEXINACA in teal, and MDMB-5’Br-BUTINACA, MDMB-4en-PINACA, and MDMB-CHMINACA in green), showing compounded inhibitory effects on both these repolarizing K_V_ channels for a limited number of SCRAs. To test the impact of compounded inhibitory effects on hERG and K_V_7.1/KCNE1 on action potential duration, we simulated the effects of applying JWH-030 or ADB-HEXINACA on human cardiomyocytes using the O’Hara-Rudy dynamic (ORd) model [41, 42] by modifying parameters for the conductance for individual channels to reflect our experimental SCRA-induced *G*_max_ effects for 10 µM of JWH-030 or ADB-HEXINACA, respectively. We saw prominent ventricular action potential prolongation induced by hERG inhibition for both SCRAs (Fig. 5k, l, denoted by *I*_Kr_). The action potential duration at 80% (APD_80_) was prolonged by 40.5 and 64.0 ms, respectively, for JWH-030 and ADB-HEXINACA. In contrast, APD_80_ was prolonged by only 5.8 and 5.5 ms, respectively, for JWH-030 and ADB-HEXINACA by K_V_7.1/KCNE1 inhibition (Fig. 5k, l, denoted by *I*_Ks_). For both SCRAs, simultaneous inhibition of hERG and K_V_7.1/KCNE1 further prolonged action potential duration compared to inhibition of hERG alone (Fig. 5k, l, denoted by *I*_Kr_ + *I*_Ks_), yielding an APD_80_ prolongation of 51.5 and 76.0 ms, respectively, for JWH-030 and ADB-HEXINACA. Hence, the impact on action potential duration of inhibiting K_V_7.1/KCNE1 was more prominent in the context of a hERG-inhibited cardiomyocyte, with the additional K_V_7.1/KCNE1 inhibition contributing by 11.0 and 12.0 ms, respectively, for JWH-030 and ADB-HEXINACA.

## Discussion

We demonstrate that the majority of SCRAs included in our study inhibit hERG, primarily by reducing the channel conductance. However, the magnitude of the effects and concentrations at which they occur varies among SCRAs. We observed important roles for both the head and tail groups. For instance, we found that several SCRAs with fluorinated alkyl tails (mostly 5F-pentyl) are prominent hERG inhibitors by reducing *G*_max_. This is interesting, as 5F-pentyl is the second most common tail moiety among SCRAs [43]. Moreover, bulky and inflexible groups seemed to impair hERG *G*_max_ effects for some SCRAs, showcased by Cumyl-TsINACA (with an inflexible, bulky tosyl tail) and 5F-APINACA (with a bulky and inflexible adamantyl head group). Interestingly, we found only partial correlation between how SCRA structural features impact the *G*_max_ effect on hERG compared to activating the CB_1_ receptor. Moreover, inhibitory SCRA effects on hERG and K_V_7.1/KCNE1 showed no apparent correlation, as fewer SCRAs had inhibitory *G*_max_ effects on K_V_7.1/KCNE1 compared to hERG, with some compounds affecting only one of the channels. These findings indicate that different chemical properties are important for SCRA binding or effects on these different targets and demonstrate the need to experimentally test the effect of SCRAs on multiple targets.

Our docking data indicate that SCRAs may use both the canonical central cavity site and the noncanonical lateral side pocket site. The SCRA orientation in the central cavity site seen in our AWH simulations is very similar to that of cryo-EM structures of the hERG pore blockers astemizole, E-4031 and pimozide [35], and the K_Ca_3.1 pore blocker 1,4-dihydropyridine [44], which all are shown to lie flat below the selectivity filter, form interaction with aromatic side chains, and are proposed to prevent ion permeation. Hence, a possibility is that SCRAs like 5F-MDMB-PINACA and 5F-BZO-POXIZID reduce *G*_max_ of hERG by obstructing and preventing currents through the pore. The direct interaction between the SCRA and the selectivity filter may also induce channel inactivation. Moreover, the SCRA pose in the lateral side pocket site seen in our docking is comparable to that recently proposed for 5F-APINACA [27], raising the possibility that SCRAs may also induce inhibiting effects from this site. The relative contribution of each site for different SCRAs cannot be deduced from our data. However, the lack of pronounced functional consequences of mutating residues S624, S649 and Y652, facing the central pore site, for 5F-MDMB-PINACA effects suggests either a greater importance of the lateral side pocket site, compensatory importance of other residues in the central cavity site or that the two sites can functionally compensate for each other. Future studies using other methods are needed to determine the relative contribution of these sites and how the interactions are impacted by channel state and structural features of SCRAs.

In modern structural biology, cryo-EM is a common tool for determining the drug-binding pocket and its interaction geometry. Information from the cryo-EM density map can be an excellent starting point for identifying the binding pose of related drug molecules, or even for understanding how they gain access to the binding site. To enrich detailed cryo-EM determined drug binding sites, computational approaches may reveal the dynamics of drug binding and drug pose adaptation independent of the side-chain rotamer for lower resolution structures. Well-tempered metadynamics recently identified a binding site of the Na_V_1.5 channel blocker flecainide in the pore just below the selectivity filter. This was very close to the site of flecainide in the cryo-EM structure, and also revealed how different compounds prefer either the central cavity or lateral fenestrations of Na_V_1.5 [45]. In our study, we used AWH simulations, which are similar to metadynamics but are native to GROMACS [46], to determine the binding of astemizole to the hERG channel with a binding mode matching the electron density map. Moreover, we extended our computational approach to four SCRAs, which showed that the shape of the free energy landscape can provide insights into the efficiency of specific SCRAs as hERG inhibitors that could not be determined from docking.

How do our findings on hERG inhibition by SCRAs compare with the current literature? Yun et al. reported JWH-030 to be an inhibitor of hERG capable of inducing QT prolongation in anesthetized rats [26]; however, their paper reports an in vitro IC_50_ of 88.4 µM (4-fold greater than our IC_50_ of 23.1 µM). Another study employed machine learning tools to predict hERG interaction of seven SCRAs [28]. Our electrophysiology data agree with their predictions for ADB-4en-PINACA and ADB-5’Br-INACA, showing no or limited effects for these SCRAs, and for 5F-BZO-POXIZID, showing prominent inhibitory *G*_max_ effects in our hERG assay. In a more recent study, Cheng et al. reported inhibitory hERG effects of 5F-APINACA with an IC_50_ of 2.2 µM [27], which is considerably lower than our IC_50_ ( > 100 µM, SI Table II). Hence, our study agrees with these reports in observing inhibitory SCRA-mediated effects on hERG, whereas experimental differences may contribute to the varying observed concentrations required to achieve effects. It is difficult to know the reason for the discrepancy in JWH-030 concentrations needed to induce hERG inhibition in the two studies, given the relatively limited information about experimental conditions in Yun et al. However, we note that their test pulse lasted for only 1 s, compared to our 5 s, which may be a contributing factor. For the discrepancy in 5F-APINACA concentrations needed to induce hERG inhibition, experimental cell systems (CHO cells in our study compared to HEK293 cells in Cheng et al.) or temperature (22 °C in our study compared to 37 °C in Cheng et al.) may contribute.

Study limitations include difficulties in comparing SCRA concentrations needed for effects across different targets, due to the use of multiple assays and SCRA delivery methods, combined with SCRAs’ high lipophilicity [47]. This also contributes to challenges in relating our findings to relevant in vivo concentrations, further complicated by a lack of accurate dosing information for SCRAs (as it is only available in most cases based on self-reports by those using SCRAs). The concentration of some SCRAs has been detected in post-mortem cases. For instance, the concentration of 5F-MDMB-PINACA in post-mortem cases is reported to be approximately 2 ng/mL ( ≈ 5.3 nM) [18–20, 48, 49] in blood or serum samples, and 1.82 ng/mL ( ≈ 4.8 nM) in the heart muscle [50]. However, it is not possible to interpret how well those concentrations reflect those reached in living individuals following SCRA use, or how they relate to the originally administered dose. Hence, with present knowledge, we cannot say whether the local SCRA concentration in the heart can reach sufficiently high concentrations for the SCRA-induced inhibition of hERG alone, or both hERG and K_V_7.1/KCNE1, to contribute to cardiotoxic effects. However, despite this caveat, we find it interesting to note that MDMB-CHMICA, which has been reported in two cases of sudden cardiac death [51, 52], and multiple analytically confirmed intoxications have reported tachycardia and ECG changes [53, 54], is one of the SCRAs showing clear inhibiting *G*_max_ effects on hERG in our study. Moreover, 5F-MDMB-PINACA is reportedly one of the most dangerous SCRAs, linked to several fatalities [18–20, 48, 49] and non-fatal intoxication with symptoms suggestive of cardiac arrhythmia (palpitations, heart pain, syncope and anxiety) [21]. We found this SCRA to be one of the most prominent in reducing *G*_max_ of hERG. Another study limitation is that the SCRA library would need to be expanded beyond our 36 SCRAs for additional SAR analysis, for instance, by performing more comprehensive analysis on SCRA molecular fingerprints.

In conclusion, our extensive functional screening of 36 SCRAs found multiple compounds with inhibitory actions on one or both repolarizing cardiac K_V_ channels. Our study lends support to SCRAs being able to use both the canonical central cavity site and the noncanonical side pocket site in hERG, highlights chemical motifs tuning the SCRA effect on hERG *G*_max_, while also providing insights into similarities and differences in SCRA features impacting CB_1_ and K_V_7.1/KCNE1 effects. Given that our screening included 13% of the 277 SCRAs that are monitored by the EUDA (formerly the EMCDDA) as of 2025 [2, 55], our study motivates the evaluation of ion channel effects of more SCRAs, as well as further exploration of SCRA interactions with different hERG binding sites and studying their cardiac effects.

## Materials and methods

### Chemicals

4F-MDMB-BUTICA, 5F-APINACA, ADB-4en-PINACA, ADB-5’Br-BUTINACA, ADB-CHMICA, ADB-FUBINACA, ADB-HEXINACA, Benzyl-4CN-BUTINACA, Cumyl-TsINACA, JWH-210, MDMB-CHMICA, MDMB-CHMINACA, MDMB-FUBINACA, and THJ-2201 reference standards (purity ≥ 98%) were purchased from Cayman Chemicals (Ann Arbor, USA). 5F-BZO-POXIZID, 5F-Cumyl-PINACA, 5F-MDMB-PINACA, AB-CHMICA, AB-CHMINACA, AB-FUBINACA, ADB-5’Br-INACA, ADB-CHMINACA, AM-2201, APINACA, BZO-POXIZID, BZO-4en-POXIZID, BZO-HEXOXIZID, JWH-018, JWH-030, JWH-122, MDMB-4en-PINACA, MDMB-5’Br-INACA, MDMB-5’Br-BUTINACA, MMB-CHMICA, UR-144, and XLR-11 reference standards (purity ≥ 98%) were purchased from Chiron AS (Trondheim, Norway). Astemizole was purchased from Sigma-Aldrich (Stockholm, Sweden). All compound solutions were in DMSO at a concentration of 10 mM and stored at –20 °C. Compounds were diluted in the extracellular recording solution on the day of recordings. DMEM/Ham’s F12, Ham’s F12, 0.25% trypsin-EDTA with phenol red, and fetal bovine serum (FBS) were purchased from Thermo Fisher (Gothenburg, Sweden). HEPES buffer, *L*-glutamine, protease-free bovine serum albumin (BSA), digitonin, adenosine-5’-triphosphate disodium salt hydrate (ATP), methanol, and DMSO were procured from Sigma-Aldrich (Stockholm, Sweden). The coelenterazine substrate was from Nanolight Technology (Pinetop, AZ, United States).

### Cell lines

Stably expressing cell lines “CHO-hERG DUO” (here referred to as hERG; gene: KCNH2) and “CHO-K_V_LQT1/mink” (here referred to as K_V_7.1/KCNE1; gene: KCNQ1/KCNE1), (B’SYS GmBH, Witterswil, Switzerland) were used to study whole-cell K^+^ currents on a QPatch II 48 APC system (Sophion Bioscience A/S, Ballerup, Denmark). Cell culture and passaging were performed in accordance with standard operating procedures. Cells were harvested in serum-free medium with Detachin^TM^ (Genlantis, CA, USA) immediately prior to experiments. For MPC on transient cell lines, CHO-K1 CCL-61 cells (ATCC, Manassas, VA, USA) were cultured in Ham’s F-12 medium supplemented with 10% fetal bovine serum (FBS) and 100 U/mL penicillin–100 µg/mL streptomycin (Gibco, Thermo Fisher Scientific, Waltham, MA, USA). Transfection was carried out using JetOPTIMUS® reagent (Polyplus, Strasbourg, France) according to the manufacturer’s protocol. Briefly, 80,000 cells were seeded per well in a 12-well plate and transfected with 300 ng of pcDNA3-GFP plasmid, 700 ng of pcDNA3-hERG plasmid with either the wild-type or mutant channels (S624A, S649A, or Y652A), and 1 µg of JetOPTIMUS. After 8 h, cells were detached and reseeded at a density of 30,000 cells per well onto several poly-*D*-lysine-coated glass coverslips placed in a 12-well plate. For the CB_1_ receptor activity assay, the AequoScreen® recombinant Chinese hamster ovary (CHO) K1 cell line, stably expressing the human CB_1_ receptor (ES-110-A), was used (Revvity, Sollentuna, Sweden).

### Constructs

The hERG wild-type and Y652A constructs were kindly provided by Dr. Nicole Schmitt, and the eGFP-pcDNA3 construct was kindly provided by Dr. Antonios Pantazis. The S624A and S649A constructs were generated in-house from the wild-type template using the QuikChange II XL Site-Directed Mutagenesis Kit (Agilent Technologies, Santa Clara, CA, USA), in accordance with the manufacturer’s instructions. Mutagenesis primers were designed using the Agilent QuikChange Primer Design Program. Briefly, PCR amplification was performed according to the recommended cycling conditions, followed by *Dpn*I digestion for 1 h to selectively degrade the parental (methylated) DNA template. The reaction products were then transformed into XL10-Gold ultracompetent cells. All constructs were verified by whole-plasmid sequencing (Eurofins Genomics, Germany) to confirm sequence accuracy.

### Automated patch-clamp experiments

Automated patch-clamp recordings were performed at 22 °C. Freshly harvested cell suspensions (2–3 million cells/mL) were prepared for experiments by the QPatch II 48 automated cell preparation unit. Cells were centrifuged for 150 s at 150 × *g* and washed twice with the extracellular recording solution. Cells were handled by the integrated pipetting robot and added to the 48 experiment sites on the planar patch-clamp consumable QPlate. Each experiment site contained an individual pair of recording electrodes and a silicon/glass biochip embedded in the flow channels. Single-cell patch recordings (*n* = 1 is defined as one single-cell patch) and population patch recordings (up to 10 cells per well, *n* = 1 is defined as one population patch) were performed for hERG and for K_V_7.1/KCNE1, respectively. Cells were applied, positioned and sealed to the patch holes by a mild suction protocol; whole-cell access was then achieved by a stronger suction pulse.

The extracellular solution contained (in mM): 145 NaCl, 10 Glucose, 4 KCl, 2 CaCl_2_, 1 MgCl_2_, and 10 HEPES. Osmolarity was adjusted to 305-308 mOsm with sucrose, and pH was adjusted to 7.4 with NaOH. The intracellular solution contained (in mM): 100 KCl, 4.3 CaCl_2_, 1.4 MgCl_2_, 25/10 KOH/EGTA, 24 KF, 10 HEPES, and 4 Mg-ATP (hERG) or 4 Na-ATP (K_V_7.1/KCNE1). Osmolarity was adjusted to 305-308 mOsm with sucrose, and pH was adjusted to 7.2 with KOH. The compounds were pre-diluted in DMSO, and the final concentration of DMSO (0.3%) was equal in all recording solutions to control for vehicle effects. The compounds made no contact with plastics following dilution in extracellular solution.

An overview of experimental conditions is provided in SI Table VII, Square voltage pulses in a voltage ladder protocol were used to study current/voltage (*I*(*V*)) relationships. Each voltage protocol was first run three times in the presence of vehicle (extracellular solution supplemented with 0.3% DMSO), the 3^rd^ serving as the baseline. Voltage protocols were repeated in the presence of 4 cumulatively rising concentrations of test compound, followed by application of vehicle for recovery. To control for assay quality, one set of vehicle-only experiments, which received no test compound, was included in each run (negative control). Each solution was added to the QPlate twice (5s between applications: 10 µL per application). Solutions were present for 60 s before recording started. hERG cells were kept at a holding voltage of −80 mV, with brief hyperpolarizing steps to −90 mV for 50 ms (to calculate ohmic leakage) before returning to −80 mV for 200 ms. Square voltage pulses were sequentially applied in 10 mV increments (range −60 mV to +60 mV) for 5 s before stepping to −50 mV for 2 s to record tail currents. Time between start of sweeps was 10 s. The voltage pulsing protocol was executed every 150 s.

For the K_V_7.1/KCNE1 voltage ladder protocol (SI Table VII) the solutions were present for 60 s before recording started. K_V_7.1/KCNE1 cells were kept at a holding voltage of −90 mV, with brief hyperpolarizing steps to −100 mV for 540 ms (to calculate ohmic leakage). Square voltage pulses were sequentially applied in 10 mV increments (range −100 mV to +40 mV) for 4 s before stepping to −20 mV for 2 s to record tail currents. The time between compound application and the first voltage protocol was 60 s. Time between sweeps was 15 s. The voltage ladder protocol was executed every 220 s.

All APC experiments on hERG were performed as described above, with the exception of experiments shown in SI Fig. S12 to monitor the onset of the SCRA effect in APC. In these specific experiments (only used for SI Fig. S12), a voltage pulsing protocol was used. The extracellular solution remained present for 7 s before recording started. hERG cells were kept at a holding voltage of −80 mV with brief hyperpolarizing steps to −90 mV for 50 ms (to calculate ohmic leakage) before returning to −80 mV. Stepping occurred in two stages, first to −50 mV for 50 ms before stepping up +40 mV for 5 s. Cells were then held −50 mV for 2 s to record tail currents and returned to −80 mV. Time between start of sweeps was 10 s, and sweeps were pulsed 20 times per protocol. The voltage pulsing protocol was executed every 201 s.

### Transient transfections and manual patch-clamp electrophysiology

Manual patch-clamp (MPC) recordings were performed 24–48 h post-transfection. Patch pipettes were pulled from borosilicate glass and had resistances of 4–7 MΩ when filled with intracellular solution. Coverslips with CHO-K1 cells were placed in a recording chamber and continuously perfused at room temperature (22–24 °C) with extracellular solution using a gravity-fed perfusion system. The intracellular and extracellular solutions used for MPC were identical to those used for APC electrophysiology (see above). Extracellular solution without DMSO was used as a vehicle. Compounds were diluted from the stock solution as a series of dilutions in extracellular solution (i.e., no compensation for different concentrations of DMSO in recording solutions). Compounds were applied to the recorded cells by a pressurized, automated OctaFlow perfusion system (ALA Scientific Instruments).

hERG cells were kept at a holding voltage of −80 mV, with brief hyperpolarizing steps to −90 mV (to calculate ohmic leakage) before returning to −80 mV. Square voltage pulses were sequentially applied in 10 mV increments (range −60 mV to +50 mV) for 5 s before stepping to −50 mV for 2 s to record tail currents (SI Table VII). Each voltage protocol was first run three times in extracellular solution, the 3^rd^ serving as the baseline. Voltage protocols were repeated in the presence of cumulatively rising concentrations of test compound; each concentration was present for 30 s before the recording started. The signals were sampled at 5 kHz after low-pass filtering at 2 kHz.

### Electrophysiological analysis

Data from APC and MPC experiments were analyzed using Sophion Analyzer 10.0 software (Sophion Biosciences, Ballerup, Denmark) and Clampfit 10.7 (Molecular Devices, San Jose, CA), respectively, and GraphPad Prism 10 (version 10.0.2; GraphPad Software Inc., CA, USA). For hERG APC-recordings, tail current was extracted as the average current from the start of the tail (70 ms after change of voltage; duration 19 ms). For hERG MPC-recordings, tail current was extracted as the peak current from the start of the tail (35–45 ms after change of voltage for WT; 20-30 ms after change of voltage for Y652A; 30–40 ms after change of voltage for S649A, 70–85 ms after change of voltage for S624A). For Kv7.1/KCNE1, tail current was extracted as the average current from the start of the tail (19.8 ms after change of voltage; duration 25.8 ms).

Ohmic leakage was subtracted from tail currents similarly for APC and MPC data, and the leak-subtracted tail currents were plotted directly against the preceding test voltages to construct *G*(*V*) curves. These data were fit with a single Boltzmann curve:

1 $$G\left(V\right)={G}_{min }+\frac{({G}_{{plateau}}-{G}_{min })}{1+{e}^{-(\frac{V-V50}{{V}_{{slope}}})}}$$

where *G*_min_ is the base, *G*_plateau_ is the top, *V* is the voltage in mV, *V*_50_ is the voltage where half of the maximum current is reached, and slope is the slope of the curve. To set the bottom of the curve to zero, the *G*_min_ was subtracted from the leak-subtracted tail currents, and data were again plotted directly against preceding test voltages and fit with a single Boltzmann curve:

2 $$G\left(V\right)=\frac{{G}_{max }}{1+{e}^{-(\frac{V-V50}{{V}_{{slope}}})}}$$

where *G*_max_ is the top, *V* is the voltage in mV, *V*_50_ is the voltage where half of the maximum current is reached, and slope is the slope of the curve. The slope was set to 8 and 12 for hERG and K_V_7.1/KCNE1, respectively. *G*_max_ and *V*_50_ values were exported from Sophion Analyzer or Clampfit (via Excel) for further analysis with GraphPad Prism.

Ratios of *G*_max_ (*G*/*G*_max_) and Δ*V*_50_ were calculated on a cell-by-cell basis and analyzed separately. To account for potential time-dependent, non–compound-related effects in APC experiments, all values were subsequently normalized to time-matched negative controls. For *G*/*G*_max_, the tail current measured in the presence of the compound was divided by the tail current obtained during the third vehicle application for each individual cell, generating a cell-specific current ratio. The mean ratio of the corresponding time-matched negative control was calculated similarly, and each individual ratio was normalized by dividing by this mean. This normalization sets the average response of the negative control to 1. Final reported values represent the mean ± SEM of the normalized ratios for each concentration. Δ*V*_50_ was calculated analogously, using differences in mV rather than ratios. For each cell, the shift in *V*_50_ induced by the compound was determined relative to the corresponding vehicle condition, and values were subsequently normalized to the mean of the time-matched negative control. This normalization sets the average of the negative control to 0. These calculations were performed in GraphPad Prism 10 using XY table format, with one compound or negative control per column and individual cells represented as subcolumns. Cell-specific calculations were performed by defining subcolumns as repeated measures, ensuring that operations were carried out on a per-cell basis. For normalization to time-matched controls, subcolumns were treated as replicates to calculate the mean control response. This mean was then used to normalize all corresponding cell-specific values. Normalized mean values and SEM were subsequently calculated for each compound and concentration. For statistical comparisons at single concentrations (e.g., 10 µM), the underlying individual cell or well measurements (subcolumns) were used directly and treated as independent observations.

For MPC recordings, ratios of *G*_max_ (*G*/*G*_max_) and Δ*V*_50_ were calculated on a cell-by-cell basis, as described for APC recordings, except that no normalization to time-matched negative controls was performed.

To determine concentration-dependence of responses to test compounds, *G*_max_ (rel.) and Δ*V*_50_ values were plotted and the following 4-parameter concentration-response curve was fitted to the data:

3 $$Y={Bottom}+\frac{({Top}-{Bottom})}{{1+({{IC}}_{50}/x)}^{N}}$$

where *Y* is the response (*G*_max_ (rel.) or Δ*V*_50_), IC_50_ is the concentration that evokes 50 % of the maximum response (constrained to always be above 0), and *N* is the Hill slope. For *G*_max_ (rel.), *N* was set to –1. For Δ*V*_50_, *N* was set to 1. For *G*_max_ (rel.), *Bottom* represents the maximally inhibited response and was constrained to assume a value between 0 and 1, and *Top* represents the baseline (no current inhibition) and was constrained to always be equal to 1. For Δ*V*_50_, *Bottom* represents the baseline (no shift in *V*_50_) and was constrained to always be equal to 0, while *Top* was the maximal *V*_50_ response and was not constrained.

Efficacy values are reported as mean ± SEM, whereas potency values are reported with asymmetrical 95% confidence intervals (CI).

Quality control criteria were applied to assess the reliability of concentration–response fits. Efficacy and potency were not determined if no clear concentration–response relationship was observed or if IC_50_ values could not be estimated with defined confidence intervals. For fits without saturation within the tested concentration range (IC_50_ > 30 µM), estimated values were considered reliable only if fit quality and parameter uncertainty met predefined criteria (*R*² > 0.8 and logIC_50_ span error < 1). We noted that some concentration–response relationships did not reach saturation within the tested concentration range; therefore, fitted parameters for hERG and Kv7.1/KCNE1 were interpreted with caution.

### In vitro biological activity at the CB_1_ receptor

The apoaequorin system (AequoScreen^®^ assay) is an intracellular calcium (Ca^2+^) release assay commonly used for measuring G-protein coupled (GPC) receptor activity. Apoaequorin is a protein that, in the presence of its substrate coelenterazine, converts to the aequorin photoprotein, which has three Ca^2+^ binding sites [56]. The binding of an extracellular ligand to the GPC receptor leads to the activation of a universal G protein subunit Gα16, which in turn triggers a series of downstream events, such as the activation of the phospholipase C (PLC) enzyme, that stimulates the release of intracellular Ca^2+^ [56]. The Ca^2+^ then binds to the aequorin photoprotein, which oxidizes the coelenterazine, leading to the release of photons that can be measured via a luminescence reader [57].

CHO-K1 cells stably expressing CB_1_, the apoaequorin enzyme, and the Gα16 subunit were maintained in a humidified atmosphere at 37 °C and 5 % CO_2_ in Ham’s F12 medium supplemented with 10 % heat-inactivated FBS. To perform the assays, cells were trypsinized (10 min, 37 °C), centrifuged (at 200 × *g*, 5 min, room temperature), counted, resuspended at 3 × 10^5^ cells/mL in DMEM/Ham’s F12 without phenol red, and supplemented with 15 mM HEPES, *L*-glutamine, and protease-free BSA (0.1%) (further referred to as assay medium). The coelenterazine substrate was added to a final concentration of 2.5 μM and the suspension was incubated for 3 h (room temperature, rotating at ~7 RPM/min, protected from light). Drug solutions were prepared as a 1:8 serial dilution in assay medium (1:4 serial dilution for Δ^9^-THC) with a starting concentration of 60 μM (in well after addition of cells) and then added to white, opaque-welled 96-well plates. JWH-018 (60 μM) was included as a reference on each plate, in line with earlier results generated using the AequoScreen^®^ CB_1_ assay [58–60]. Digitonin (67 μM) and ATP (6.7 μM) were included on each plate and served as positive controls for coelenterazine loading, as both are involved in the non-CB-dependent release of calcium ions. Blank assay medium was used as a negative control. Using a TECAN Spark 10 M plate reader (Männedorf, Switzerland), 50 μL of the incubated cell suspension was dispensed into each well (15 × 10^3^ cells/well) of the 96-well plate containing the test solutions. Luminescence was measured for 25 s (corresponding to 190 additional reading cycles).

Absolute luminescence signals were corrected for intra-plate variability in Microsoft Excel 365 using area under the curve (AUC) values of JWH-018 and were calculated for each concentration of the test compounds. Values were then blank-corrected by subtracting the AUC values of the mean of the blank controls. Data were normalized to the maximum of JWH-018. The normalized values were transferred to GraphPad Prism (Version 10.0.2) to generate concentration-response curves and calculate EC_50_ and *E*_max_ values by curve fitting via nonlinear regression (three-parameter logistic fit). Results are represented as receptor activity of JWH-018 (%) derived from a minimum of three independent biological replicates (*n* ≥ 3), run in triplicate (technical replicates). There were only two independent experiments (*n* = 2) for JWH-030, as there was not enough of the reference standard available for a third independent experiment. As such, JWH-030 was not included in any statistical analysis for SARs. As the CB_1_ receptor activity of the SCRAs included in this study was examined over the span of a couple of years, EC_50_ values were normalized to the median EC_50_ value of JWH-018 (35.03 nM) from 139 independent experiments run over many years to adjust for the slight variability in receptor activity over time (13.7-51.9 nM). There was no statistically significant difference between the EC_50_ values before and after normalization (ANOVA, α = 0.05; *P* = 0.89).

### SAR-studies

Structure-activity relationship (SAR) analysis for the CB_1_ receptor was conducted for both efficacy (*E*_max_) and potency (EC_50_), where the logEC_50_ values were used for the statistical analysis. Only comparisons involving at least three pairs of structurally related compounds were included. The compounds in each pair were evaluated using Brown-Forsythe and Welch ANOVA tests (α = 0.05), and the overall comparison (all pairs with the same structural change) was statistically evaluated using a paired *t*-test (α = 0.05).

### Statistical analysis

Data are reported as mean ± SEM. GraphPad Prism 10 was used for all statistical analyses. *n* = 1 is defined as one single-cell patch for hERG; one population patch for Kv7.1/KCNE1, one biological replicate (calculated as an average of three technical replicates) for CB_1_. For CB_1_ overall SAR comparisons, *n* = 1 is defined as one pair of compounds. A significance level of *P* < 0.05 was used for all statistical tests. Statistical significance is indicated as follows: *P < 0.05, **P < 0.01, ***P < 0.001, and ****P < 0.0001.

For comparisons at single concentrations (e.g., 10 µM), individual cell or well measurements were used as independent observations. Unpaired comparisons involving a single compound versus its time-matched vehicle control were performed using Welch’s *t*-test. For comparisons involving multiple compounds sharing the same time-matched vehicle control, one-way ANOVA (no matching) was applied, followed by Dunnett’s multiple comparisons test versus the vehicle control. Homogeneity of variance was assessed using the Brown–Forsythe test, and when significant heteroscedasticity was detected (*P* < 0.05), Welch’s ANOVA followed by Dunnett’s T3 multiple comparisons test was used instead.

For comparisons of IC_50_ values for 5F-MDMB-PINACA recorded using MPC on WT and mutated versions of hERG, an Extra-sum-of-squares *F*-test was used (*P* < 0.05) required for significance. For pairwise comparisons, one-way ANOVA with Šídák’s multiple comparisons test was used.

For SAR studies on CB_1_, *P* values for compound-to-compound comparisons were obtained using Brown-Forsythe and Welch ANOVA tests, while *P* values for overall comparisons were calculated using two-tailed paired t-tests.

### Molecular docking

To determine an initial binding pose for astemizole, we used the hERG channel structure with astemizole density, but no structure of astemizole was built in (PDB ID: 7CN1, [32]) and on the structure with astemizole density, and with astemizole built in afterward (PDB ID: 8ZYO) from the RCSB protein data bank. We then used Autodock Tools to dock astemizole to get the initial binding pose, selecting a grid box of 24 Å centred on residue Y652 in the vestibule of the hERG channel. Autodock Vina [61] was utilized to calculate the docking of astemizole within the box. Twenty independent docking runs were attempted for each ligand, and 15 poses were generated for each run. Outputs were clustered based on the root-mean square deviation (RMSD), and the initial binding pose was used to guide the AWH simulations. The docking was then extended to all 36 SCRAs on both structures (PDB entries: 7CN1 and 8ZYO) with a grid box of 20 Å centred on residue Y652 in the central cavity of the hERG channel, denoted as the central cavity site, and docking centered at F557 for the side pocket site.

### System set-up for molecular dynamics simulations

The 7CN1 hERG channel structure (PDB entry: 7CN1, [32]) from residue T399-L666 was embedded in a 100 % POPC bilayer and solvated in 0.15 KCl and TIP3P water using CHARMM-GUI Membrane Builder. The protein, lipids and ions were simulated using CHARMM36m forcefield with TIP3P water model. A single ligand (SCRAs) was aligned with the binding pose of astemizole at the minima from the free energy landscape obtained from AWH using PyMOL to generate an initial configuration. For the first AWH simulation, the position of astemizole was randomly placed in the central cavity. Ligand parameters were generated using Ligand Reader and Modeler on CHARMM-GUI with the CGENFF force field. The system was then energy-minimized and equilibrated using the standard six-step CHARMM-GUI equilibration protocol [62]. This includes the following set-up: protein backbone was restrained at the force constant of 4000, 2000, 1000, 500, 200 and 50 kJ·mol^−1^ ·nm^−2^, protein side chains were restrained at the force constant of 2000, 1000, 500, 200, 50, and 0 kJ·mol^−1^ ·nm^−2^, non-H atoms of lipids were restrained at the force constant of 1000, 400, 400, 200, 40 and 0 kJ·mol^−1^ ·nm^−2^, and dihedral restraints were set at the force constant of 1000, 400, 200, 200, 100 and 0 kJ·mol^−1^ ·rad^−2^. Simulations were equilibrated with a 1 fs timestep for 125 ps for the first three steps, and then to a 2 fs timestep for 500 ps the next two, and 5 ns for the final step. The first two steps were conducted with the NVT ensemble, and the last four steps were conducted with the NPT ensemble. All equilibration runs were conducted at 310 K using a Berendsen thermostat [63]. In all NPT ensemble equilibration, the pressure was maintained at 1 bar using a Berendsen barostat [63]. All simulations were conducted using GROMACS-2023.4 [64]. All images from simulations were generated using PyMOL.

### Accelerated weighted histogram simulations

The free energy landscape describing the path (z-axis distance) of the drug entering the binding site within the hERG channel was calculated using an accelerated weighted histogram (AWH) [65]. Pressure was maintained at 1 bar and temperature was maintained at 310 K. An independent AWH bias was applied for each equilibrated structure, simulating 4 walkers for 1250 ns per walker, sharing bias data and target distribution. Bias acted on the z-axis, defined using the center-of-mass of the drug and the Y652 residue in the vestibule of the hERG channel, aiming for a converged simulation, defined as a similar free energy landscape every 50 ns. Sampling interval was 1.0 nm above Y652 and 3.5 nm below Y652. The system initialized with an average free energy error of 20 kJ/mol, with the diffusion coefficient at 0.0002 nm^2^/ps at a force constant of 12800 kJ· mol^−1^ ·nm^−2^. A harmonic potential was applied to all Cα atoms at 5.0 kJ· mol^−1^ ·nm^−2^. Free energy landscapes from 1210, 1220, 1230, 1240 and 1250 ns were averaged and used to build a final free energy landscape. The binding pose was clustered using DBSCAN with an epsilon of 0.2 and a minimum sample of 10. From each cluster, 1000 structures located closest to the free energy minima were extracted and used for minimum distance calculation.

### ORd Model

MATLAB simulations of the ventricular action potential in the epicardium of the human heart were performed using the ORd model as previously described [41, 42]. To simulate the effects of SCRAs, we altered the conductance of *I*_Kr_, *I*_Ks_, or both simultaneously in the MATLAB code, similarly to what was found experimentally for 10 µM of the SCRAs in APC experiments (by multiplying the *I*_Kr_ or *I*_Ks_ conductance by the factor decrease we observed in our experiments for *G*_max_).

## Supplementary information

Supplementary information.

## Data Availability

All data related to experimental findings are provided in the main manuscript or the Supplementary Information. Trajectories and data related to molecular dynamics findings are found on Zenodo (https://doi.org/10.5281/zenodo.20717473).
