# Supplementary material for: Synthetic cannabinoid receptor agonists inhibit the cardiac voltage-gated potassium channel hERG
Source: Acta Pharmacol Sin. 2026 Jul 21;47(10):2940–54. doi: 10.1038/s41401-026-01876-9 (PMC13586173; doi:10.1038/s41401-026-01876-9)
Supplement: Supplementary file 1 [file 41401_2026_1876_MOESM1_ESM.pdf]

Supplementary Information

Synthetic cannabinoid receptor agonists inhibit the cardiac voltage-gated potassium channel hERG

Nina E. Ottosson[1,2*], Damon J. A. Frampton[1*], Tanadet Pipatpolkai[3*#], Caitlyn Norman[1], Urban Karlsson[1], Amaia Jauregi-Miguel[1,2], Maryke Venter[1], Akshay Sridhar[1], H. Peter Larsson[1], Henrik Gréen[1,4], Sara I. Liin[1#]

[1] Department of Biomedical and Clinical Sciences, Linköping University, Linköping, Sweden
[2] Chemical Biology Consortium Sweden, Science for Life Laboratories, Linköping University, Linköping, Sweden
[3] School of Physical and Mathematical Sciences, Nanyang Technological University, Singapore
[4] Department of Forensic Genetics and Forensic Toxicology, National Board of Forensic Medicine, Linköping, Sweden

[*] Indicates shared authorship
[#] Indicates corresponding authors: T.P.: tanadet.pipatpolkai@ntu.edu.sg, S.I.L.: sara.liin@liu.se

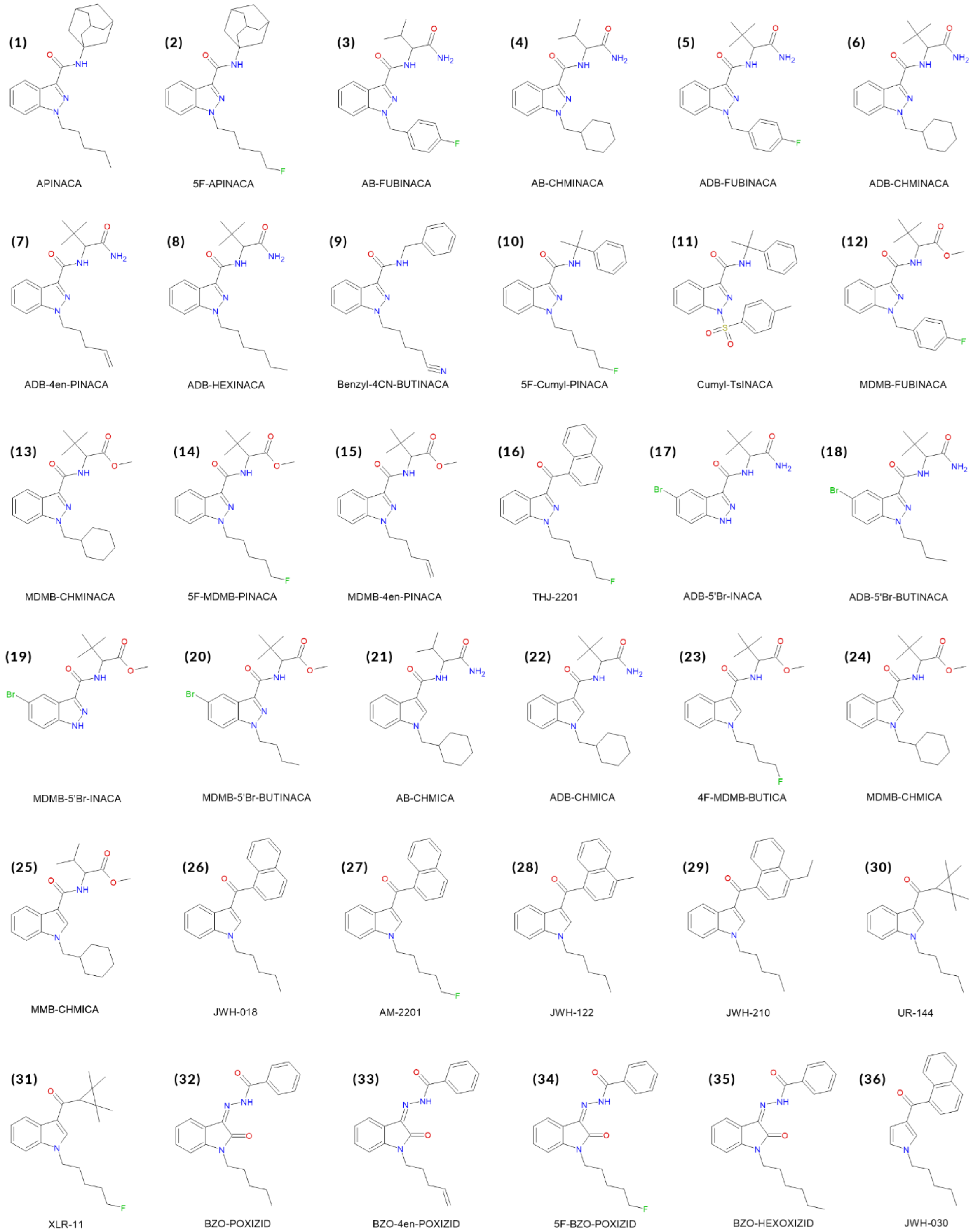

Supplementary Figure S1. Molecular structures of the SCRAs included in this study. SCRAs are numbered according to the list order in Supplementary Table I.

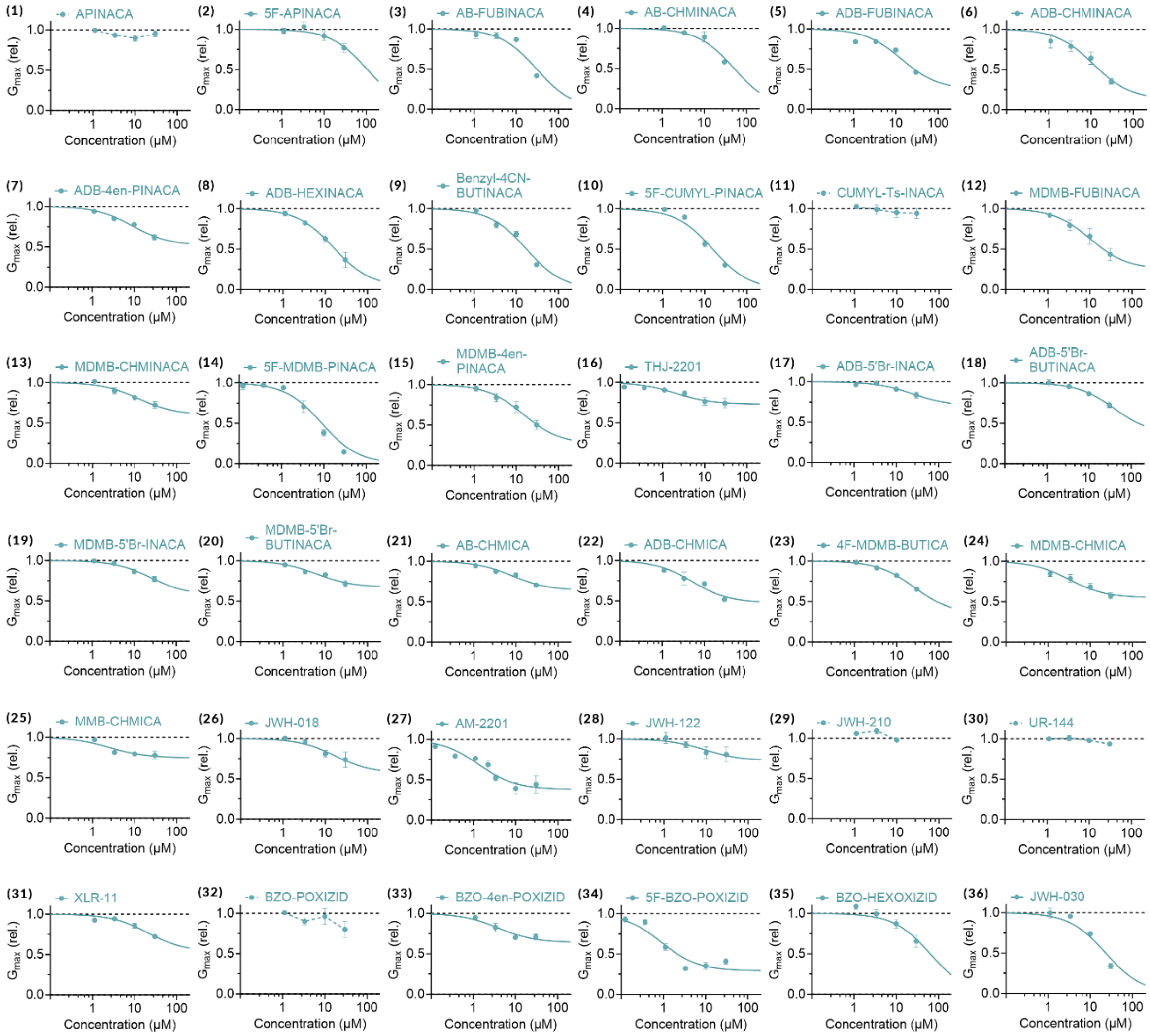

Supplementary Figure S2. Concentration-response relationships for SCRA effects on G_{max} of hERG. All average data are shown as mean ± SEM. Small error bars are covered by symbols. n = 3-13. Best fits as in SI Table II, quality control as in SI Table III. SCRAs for which no concentration–response fit could be obtained (reported as ND in SI Table II) are shown with data points connected by dashed lines. SCRA numbering is according to Supplementary Figure S1 and Supplementary Table I.

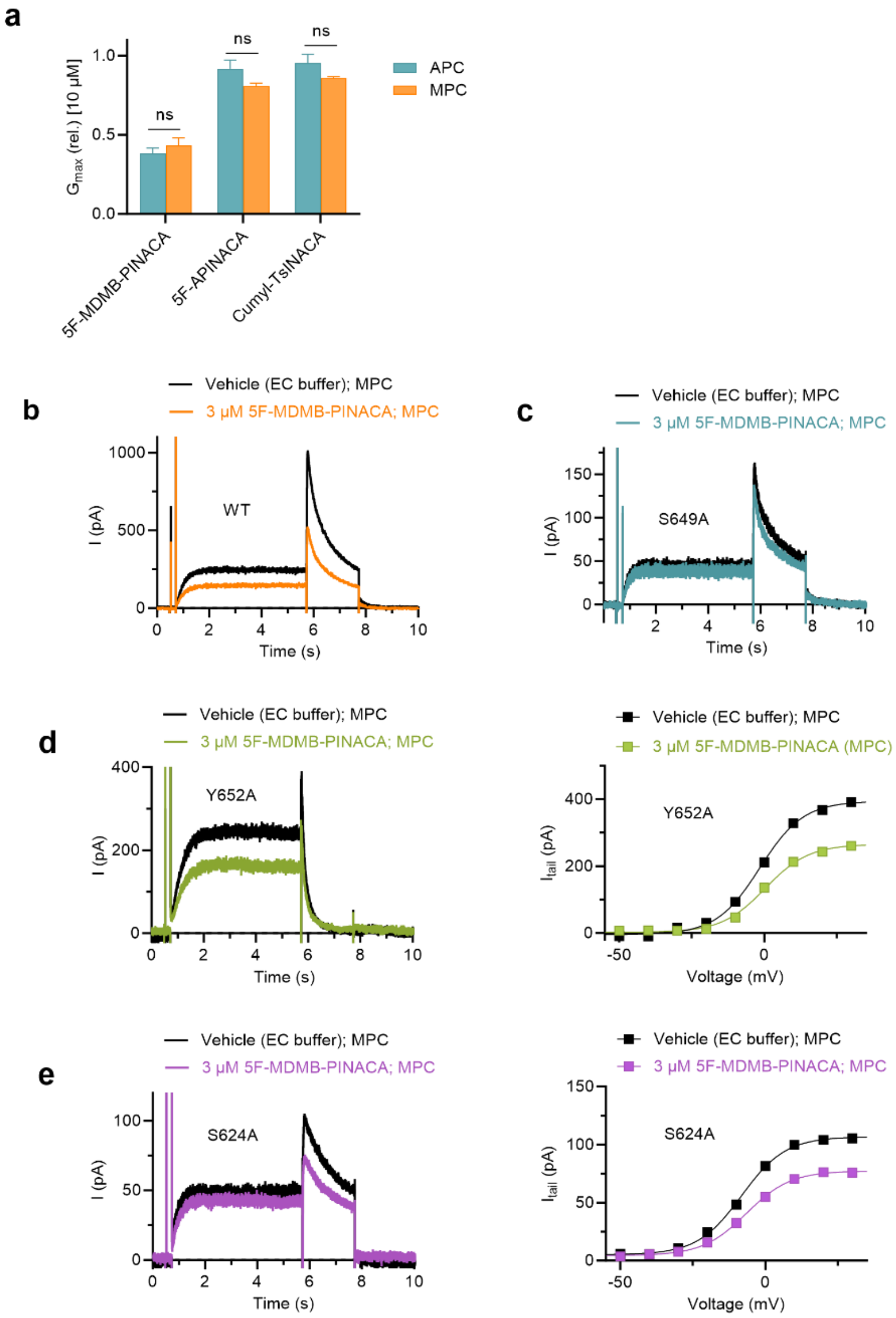

Supplementary Figure S3. Effects of SCRAs on hERG WT measured using APC and MPC, and on mutant hERG channels measured using MPC. (a) Comparison of effect of 10 µM of indicated SCRAs on hERG using APC and MPC. n = 13, 20, 12, 6, 7 and 5, respectively. Data shown as mean ± SEM. Statistics denote one-way ANOVA with Šídák's multiple comparisons test. (**b-e**) Representative current traces from MPC recordings for hERG WT (b), S649A (c), Y652A (d), and S624A (e) in the presence of 3 µM 5F-MDMB-PINACA. Cells were depolarized to +30 mV, and tail currents were recorded at −50 mV. Corresponding G(V) relationships are shown for Y652A (d) and S624A (e). Data shown as mean ± SEM.

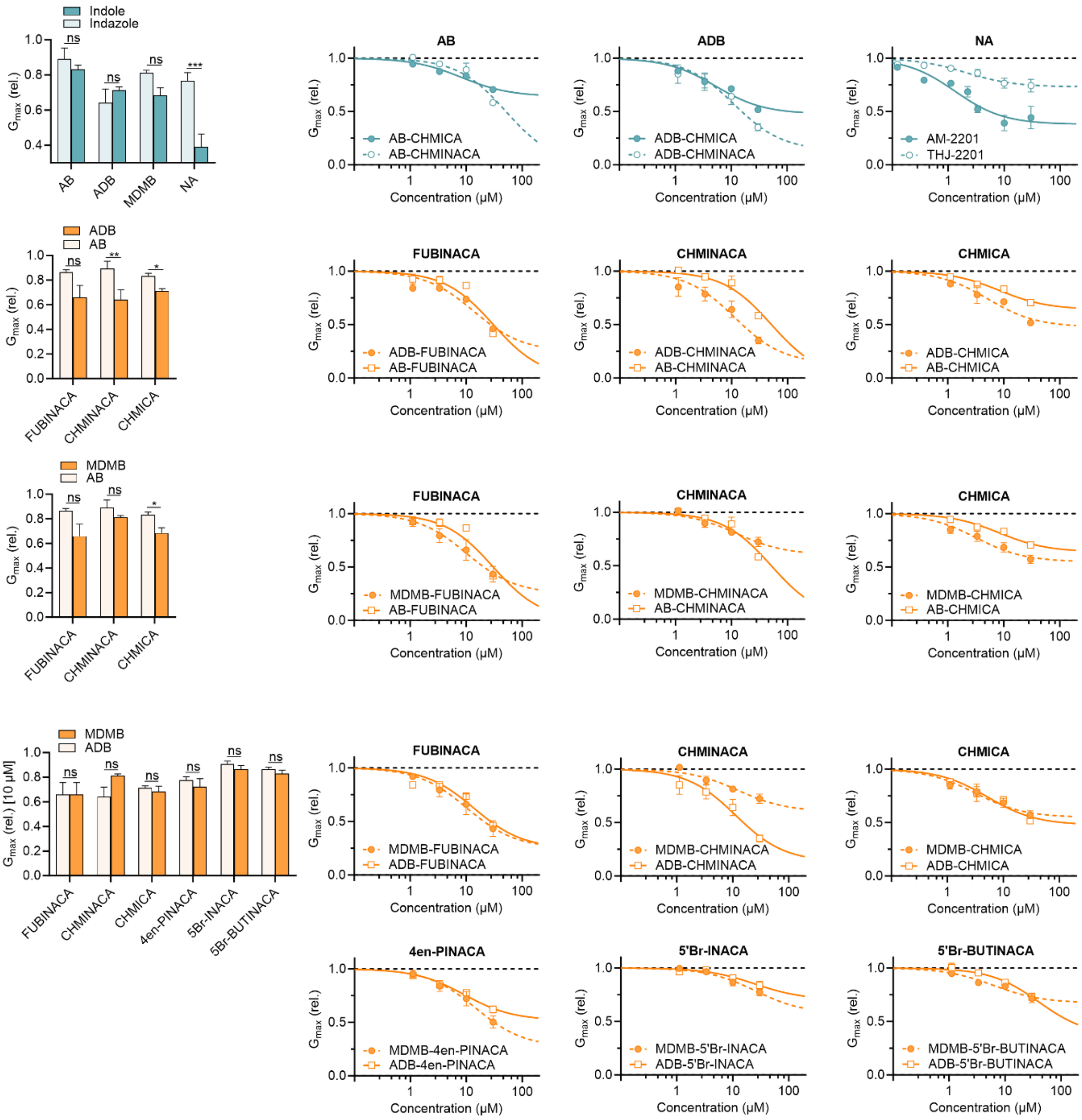

Supplementary Figure S4. Chemical modifications to the SCRA building blocks impact hERG effects. Comparison of effect at 10 µM (left) and additional concentrations (right) for G_{max} reduction in hERG for indicated structural features. Data shown as mean ± SEM. n = 4-9. Statistical comparisons were performed using one-way ANOVA followed by Šídák's multiple comparisons test. Homogeneity of variance was assessed using the Brown–Forsythe test, and no significant heteroscedasticity was detected. Statistical annotations denote adjusted p-values for comparisons to vehicle: *P < 0.05, **P < 0.01, ***P < 0.001, and ****P < 0.0001.

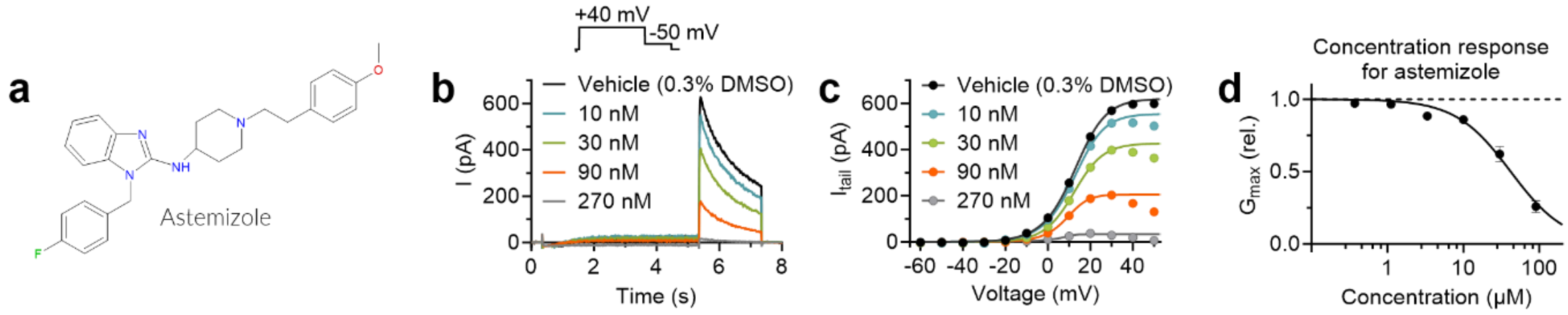

Supplementary Figure S5. Effect of the hERG blocker astemizole in the APC hERG assay. (a) Molecular structure of astemizole. **(b-c)** Representative effect of astemizole on hERG using the voltage protocol shown as insert in b, with corresponding G(V) curves in c. **(d)** Concentration-response relationships for astemizole on hERG inhibition. Best fit indicates IC_{50} = 41.9 nM, 95% CI = 32.7-47.9. n = 4. Data shown as mean ± SEM. Small error bars are covered by symbols.

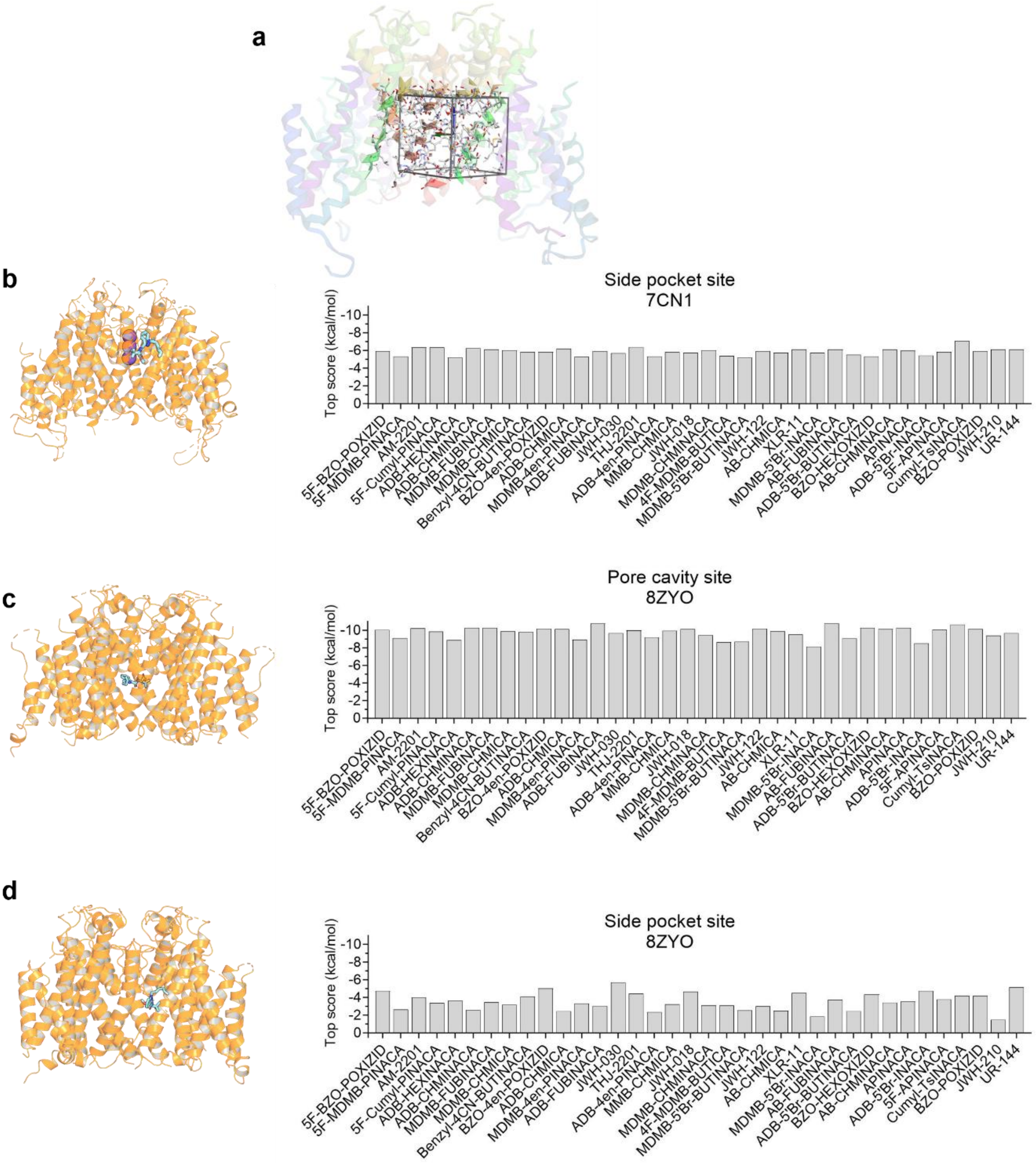

Supplementary Figure S6. Summary of hERG docking scores for SCRAs. (a) The docking grid box at 20 Å centred around Y652 on hERG structure (PDB entry: 7CN1) shown as coloured ribbons. **(b-d)** Left – representative binding pose of astemizole after docking onto (b) 7CN1 structure using Y652 as the grid centre. (c) 8ZYO using Y652 as the grid centre. (d) 8ZYO using F557 as the grid center. Right – the top docking score for all 36 SCRAs. The binding pose is shown to be located at the non-canonical side pocket in (b) and (d).

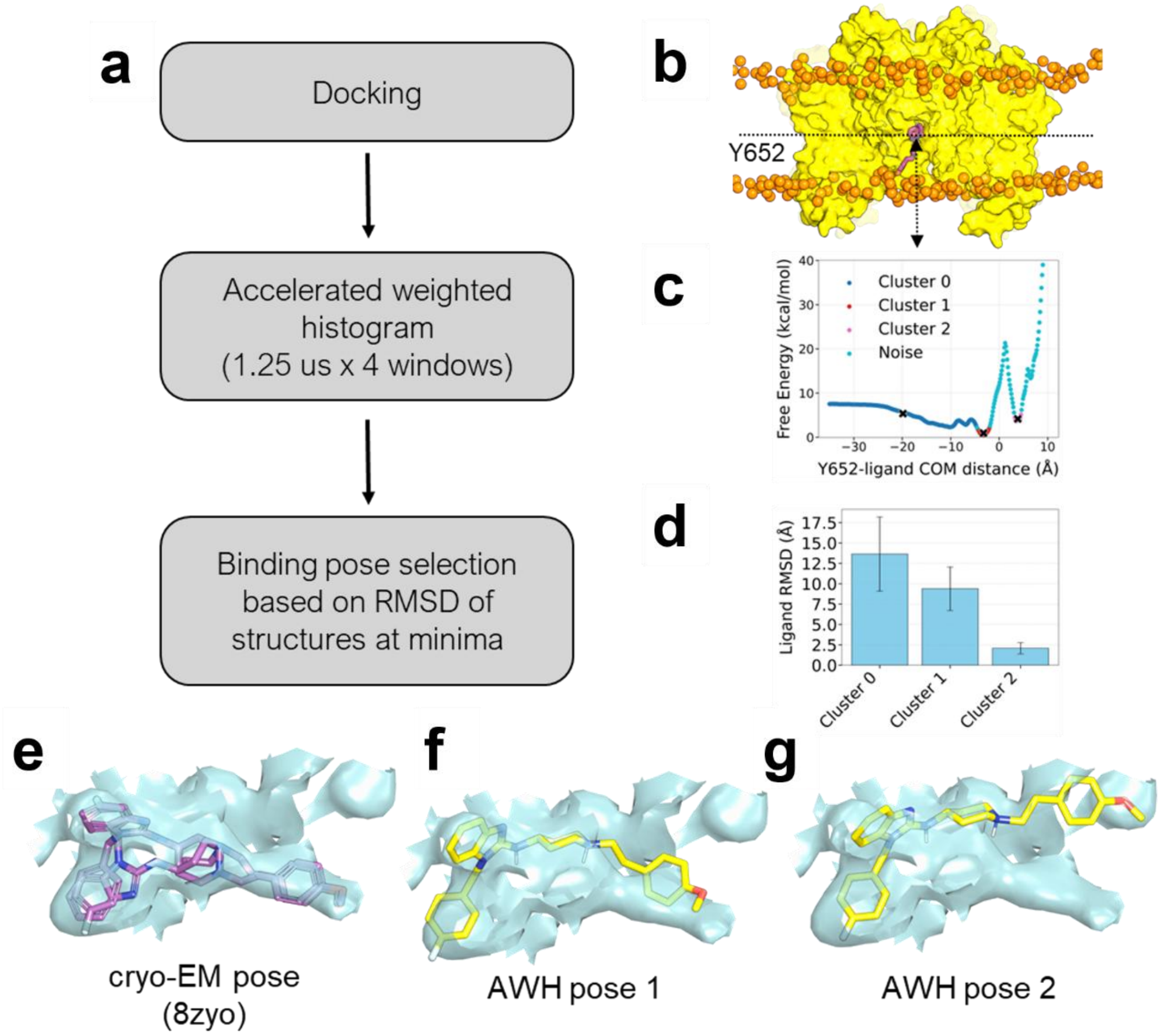

Supplementary Figure S7. AWH workflow used to determine SCRA binding. (a) *In silico* workflow employed to obtain the binding pose of astemizole and SCRAs. **(b)** The structural representation of the sampling of the astemizole ligand along the z-axis with respect of Y652. **(c)** Free energy landscape of astemizole from AWH simulations using 4 windows simulating for 1250 ns. The region highlighted with the cross shows the energy minima from each cluster. **(d)** Root mean square deviation (RMSD) of the ligand within the cluster, calculated from 1000 ligands in the binding site calculated with respect to the cluster centre. The error bar shows the standard deviation of the RMSD. **(e-g)** The electron density map (cyan) of the ligand binding site, fitting around the ligand astemizole. The cryo-EM model (PDB entry: 8ZYO) is shown in purple (e). The frames from Cluster 1 from AWH simulations in two distinct conformations are shown in yellow (f-g).

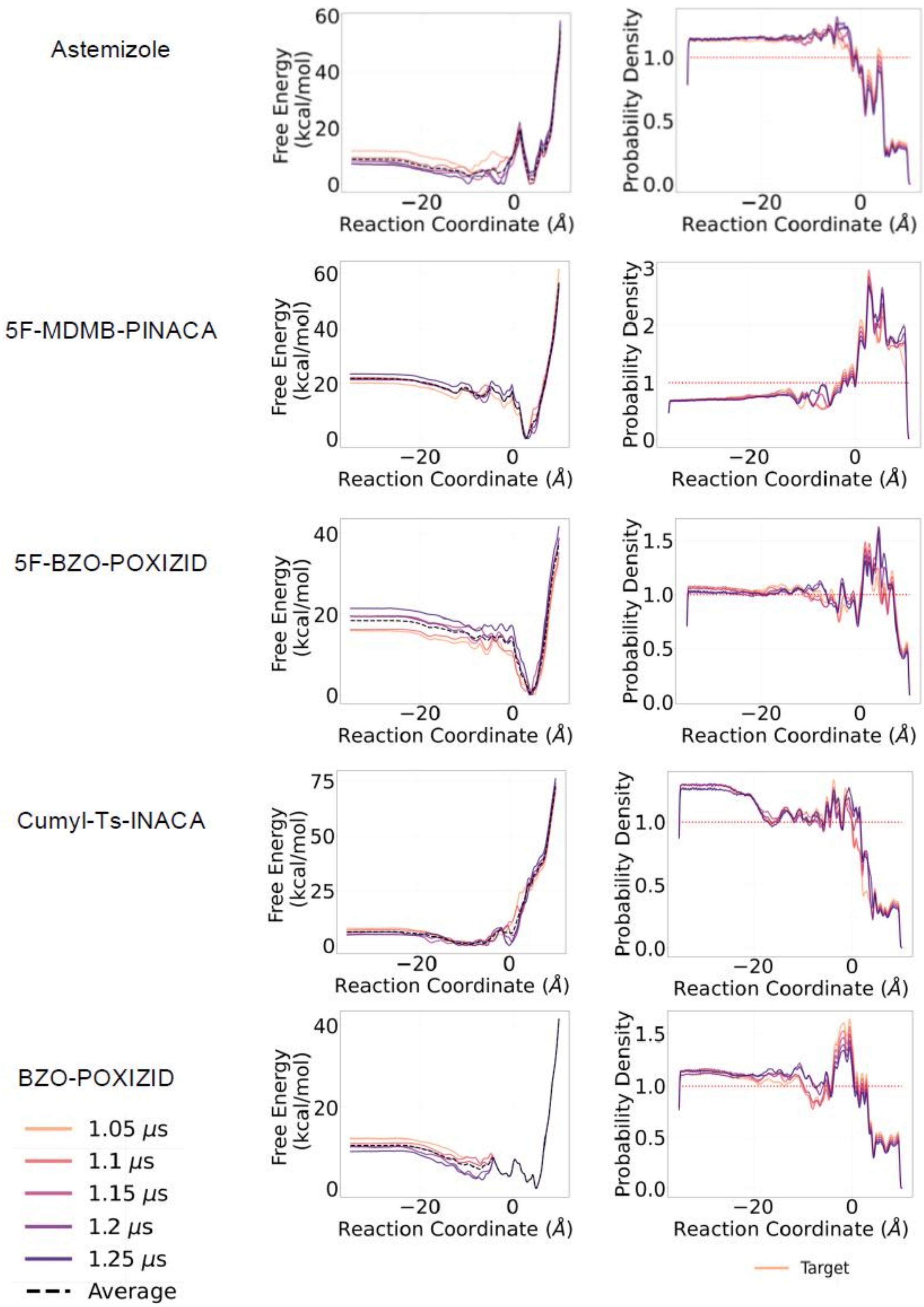

Supplementary Figure S8. Convergence plots for AWH simulations. The convergence of AWH simulations for the sampling of astemizole, 5F-MDMB-PINACA, 5F-BZO-POXIZID, BZO-POXIZID and Cumyl-TsINACA along the z-axis on the 7CN1 PDB structure, with each drug starting location at the central pore cavity. Left – The free energy landscape projecting along the z-axis centre of mass distance between the drug and Y652 at 1.05, 1.10, 1.15, 1.20, and 1.25 μs are shown in different colours. The average free energy landscape is shown as a dashed line. Right – The coordinate distribution of the sampling with the target being the dashed red line. The distribution at 1.05, 1.10, 1.15, 1.20, and 1.25 μs are shown in different colours.

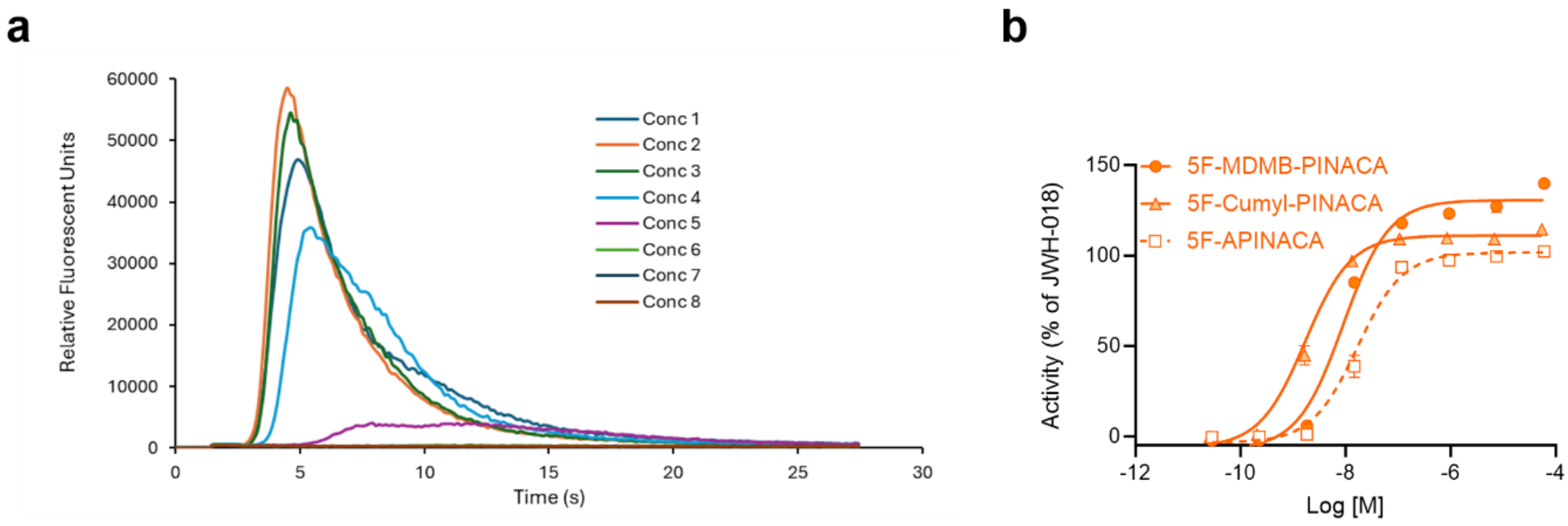

Supplementary Figure S9. CB_1 receptor activity assay. (a) Representative effect of JWH-018 on CB_1 receptor activity. The fluorescence signal of 8 concentrations of JWH-018 (n = 1) was recorded over time on the AequoScreen® CB_1 receptor assay. **(b)** Example of impact of head chemistry for CB_1 activation showcased by 5F-PINACA compounds with either MDMB (n = 4), Cumyl (n = 4), or adamantyl (n = 3) heads. All average data are shown as mean ± SEM. Small error bars are covered by symbols. Best fits as in SI Table II.

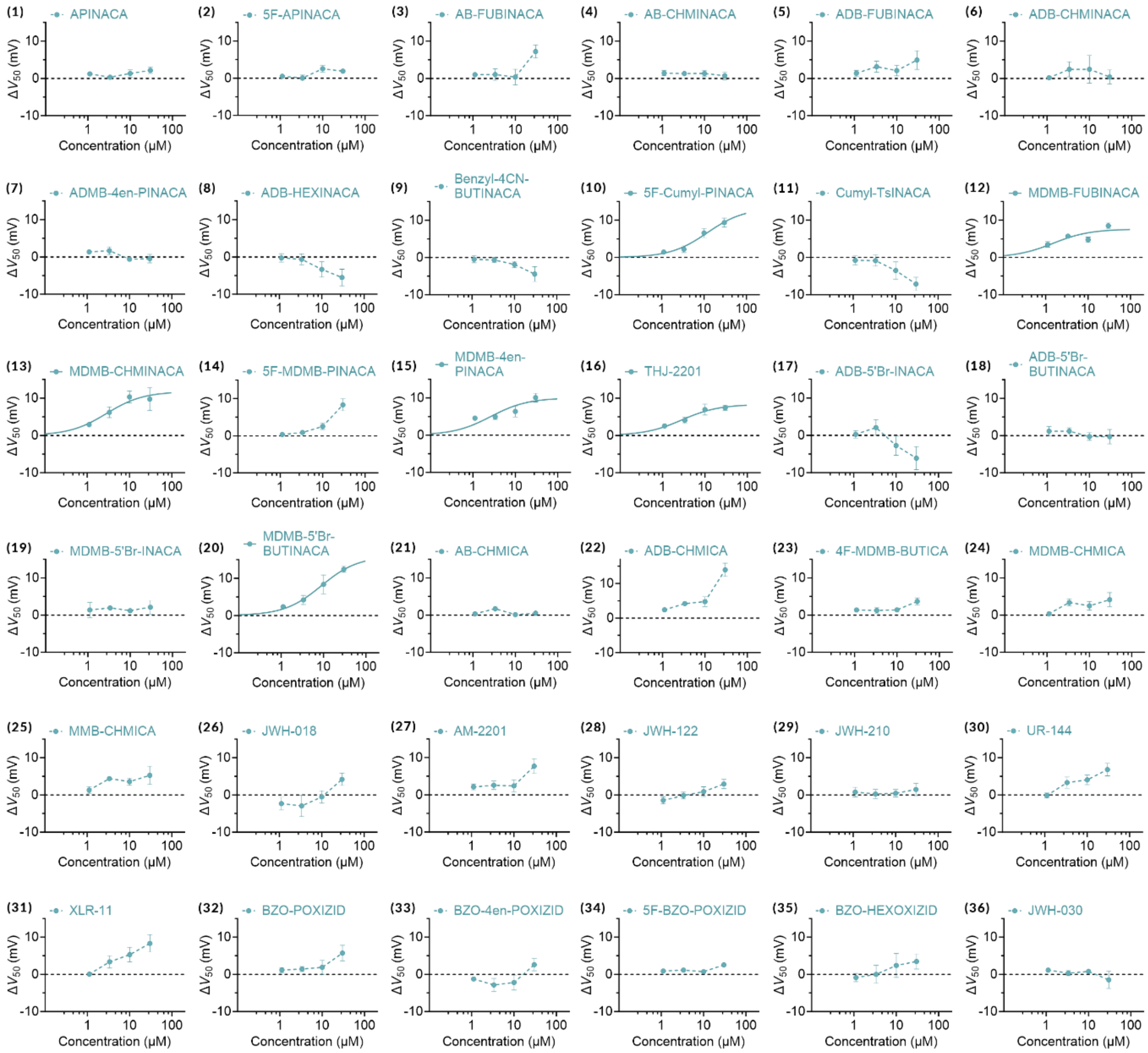

Supplementary Figure S10. **Concentration-response relationships for SCRA effects on V_{50} of Kv7.1/KCNE1.** All average data are shown as mean ± SEM. Small error bars are covered by symbols. n = 3-9. Only SCRAs meeting the predefined fit quality criteria are shown with fitted concentration–response curves. Fits were accepted only when a clear concentration–response relationship was observed and IC_{50} values could be estimated with defined confidence intervals. SCRAs not fulfilling these criteria are shown with data points connected by dashed lines and without curve fitting. SCRA numbering is according to Supplementary Figure S1 and Supplementary Table I.

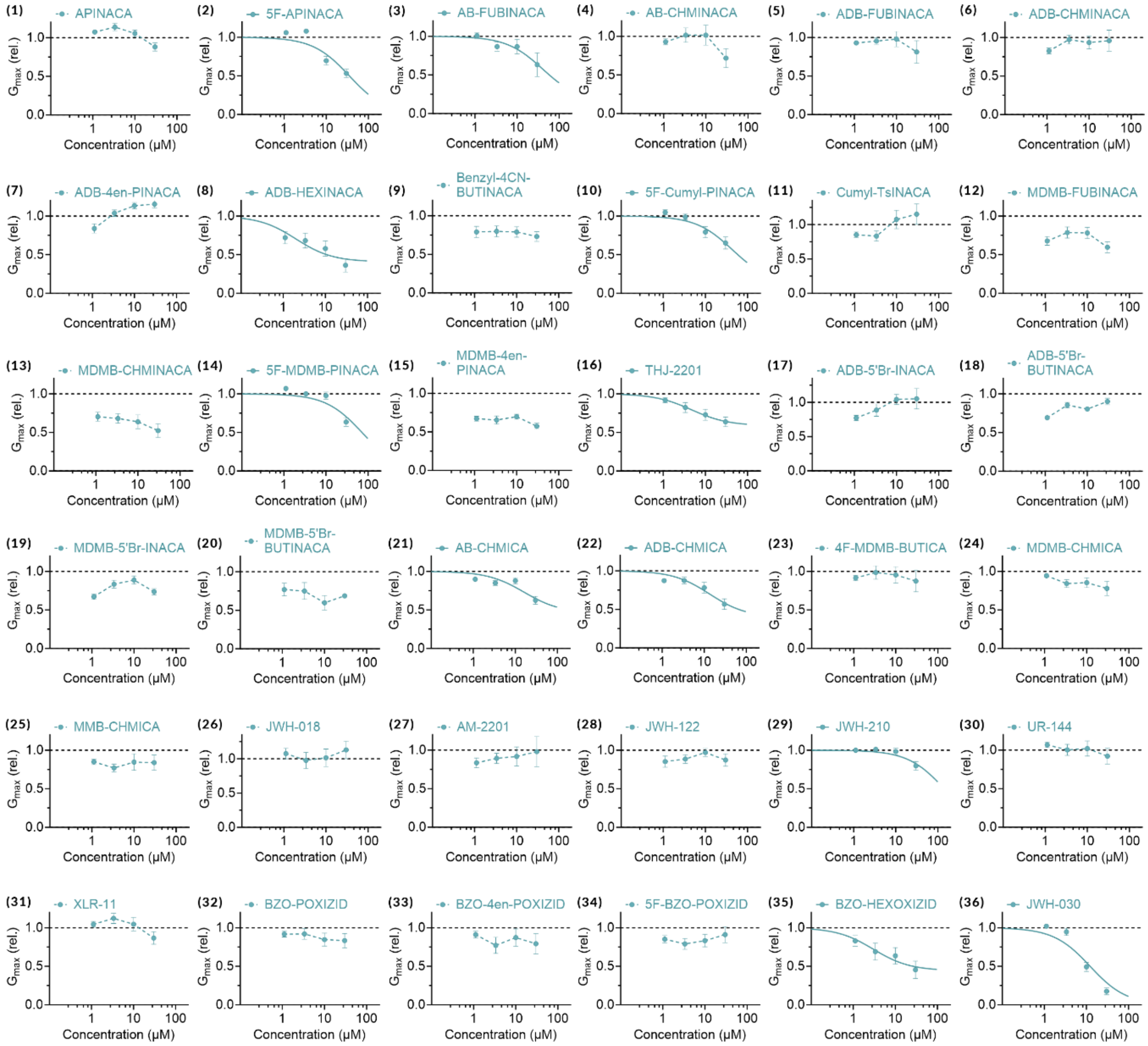

Supplementary Figure S11. **Concentration-response relationships for SCRA effects on G_{max} of Kv7.1/KCNE1.** All average data are shown as mean ± SEM. Small error bars are covered by symbols. n = 3-9. Best fits as in SI Table II. Only SCRAs meeting the predefined fit quality criteria are shown with fitted concentration–response curves. Fits were accepted only when a clear concentration–response relationship was observed and IC_{50} values could be estimated with defined confidence intervals. SCRAs not fulfilling these criteria are shown with data points connected by dashed lines and without curve fitting. SCRA numbering is according to Supplementary Figure S1 and Supplementary Table I.

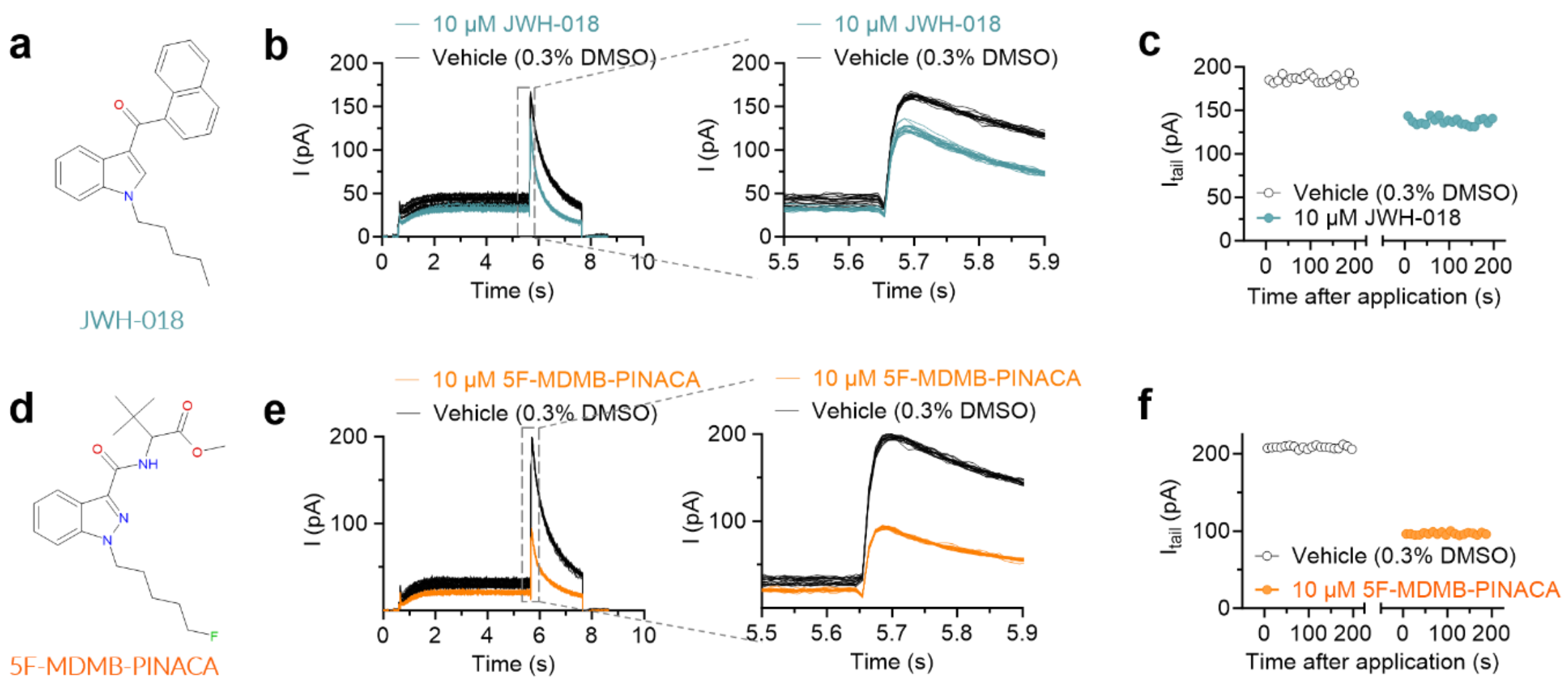

Supplementary Figure S12. Rapid onset and stable inhibition of hERG currents revealed by pulsed protocol. (a, d) Chemical structures of JWH-018 (a) and 5F-MDMB-PINACA (d). **(b, e)** Representative current traces recorded using a pulsed protocol (20 repetitive pulses) to assess the time course of compound effects. Currents were elicited by repeated depolarizing pulses (activation step to +40 mV), allowing high temporal resolution of drug action. Insets show magnified views of the onset of inhibition following compound application. **(c, f)** Time course of tail current following compound application.

Supplementary Table I: The 36 SCRAs included in this study organized by their four main structural components (core, linker, head group, and tail). For schematic example, see Figure 1c. Molecular structures of all SCRAs are shown in Supplementary Figure S1.

	SCRA name	Other used name(s)	Core	Linker	Head	Tail
1	APINACA	A-PINACA, AKB-48	Indazole	Carboxamide	Adamantyl	Pentyl
2	5F-APINACA	A-5F-PINACA, 5F-AKB-48	Indazole	Carboxamide	Adamantyl	5-fluoropentyl
3	AB-FUBINACA	AMB-FUBINACA	Indazole	Carboxamide	AB	4-fluorobenzyl
4	AB-CHMINACA	AMB-CHMINACA	Indazole	Carboxamide	AB	Cyclohexylmethyl
5	ADB-FUBINACA	ADMB-FUBINACA	Indazole	Carboxamide	ADB	4-fluorobenzyl
6	ADB-CHMINACA	ADMB-CHMINACA	Indazole	Carboxamide	ADB	Cyclohexylmethyl
7	ADB-4en-PINACA	ADMB-4en-PINACA	Indazole	Carboxamide	ADB	Pent-4-enyl
8	ADB-HEXINACA	ADMB-HINACA	Indazole	Carboxamide	ADB	Hexyl
9	Benzyl-4CN-BUTINACA	BZ-4CN-BINACA	Indazole	Carboxamide	Benzyl	4-cyanobutyl
10	5F-Cumyl-PINACA	CUMYL-5F-PINACA	Indazole	Carboxamide	Cumyl	5-fluoropentyl
11	Cumyl-TsINACA	CUMYL-TSINACA	Indazole	Carboxamide	Cumyl	Tosyl
12	MDMB-FUBINACA		Indazole	Carboxamide	MDMB	4-fluorobenzyl
13	MDMB-CHMINACA		Indazole	Carboxamide	MDMB	Cyclohexylmethyl
14	5F-MDMB-PINACA	MDMB-5F-PINACA, 5F-ADB	Indazole	Carboxamide	MDMB	5-fluoropentyl
15	MDMB-4en-PINACA		Indazole	Carboxamide	MDMB	Pent-4-enyl
16	THJ-2201	NA-5F-PINAMO	Indazole	Methanone	1-naphthyl	5-fluoropentyl
17	ADB-5'Br-INACA	ADMB-5Br-INACA	5-bromo-1H-indazole	Carboxamide	ADB	-
18	ADB-5'Br-BUTINACA	ADMB-B-5Br-INACA	5-bromo-1H-indazole	Carboxamide	ADB	Butyl
19	MDMB-5'Br-INACA	MDMB-5Br-INACA	5-bromo-1H-indazole	Carboxamide	MDMB	-
20	MDMB-5'Br-BUTINACA	MDMB-B-5Br-INACA	5-bromo-1H-indazole	Carboxamide	MDMB	Butyl
21	AB-CHMICA	AMB-CHMICA	Indole	Carboxamide	AB	Cyclohexylmethyl
22	ADB-CHMICA	ADMB-CHMICA	Indole	Carboxamide	ADB	Cyclohexylmethyl
23	4F-MDMB-BUTICA	MDMB-4F-BICA	Indole	Carboxamide	MDMB	4-fluorobutyl
24	MDMB-CHMICA		Indole	Carboxamide	MDMB	Cyclohexylmethyl
25	MMB-CHMICA	AMB-CHMICA	Indole	Carboxamide	MMB	Cyclohexylmethyl
26	JWH-018	NA-PIMO	Indole	Methanone	1-naphthyl	Pentyl
27	AM-2201	NA-5F-PIMO, 5F-JWH-018	Indole	Methanone	1-naphthyl	5-fluoropentyl
28	JWH-122	4Me-NA-PIMO	Indole	Methanone	4-methyl-naphthalen-1-yl	Pentyl
29	JWH-210	4Et-NA-PIMO	Indole	Methanone	4-ethyl-naphthalen-1-yl	Pentyl
30	UR-144	TMCP-PIMO	Indole	Methanone	Tetramethylcyclopropyl	Pentyl
31	XLR-11	TMCP-5F-PIMO, 5F-UR-144	Indole	Methanone	Tetramethylcyclopropyl	5-fluoropentyl
32	BZO-POXIZID	5C-MDA-19	2-oxindole	Hydrazide	Benzoyl	Pentyl
33	BZO-4en-POXIZID		2-oxindole	Hydrazide	Benzoyl	Pent-4-enyl
34	5F-BZO-POXIZID	BZO-5F-POXIZID, 5F-MDA-19	2-oxindole	Hydrazide	Benzoyl	5-fluoropentyl

	SCRA name	Other used name(s)	Core	Linker	Head	Tail
35	BZO-HEXOXIZID	BZO-HOXIZID, MDA-19	2-oxindole	Hydrazide	Benzoyl	Hexyl
36	JWH-030	NA-PPYOMO	1H-pyrrole	Methanone	1-naphthyl	Pentyl

*Abbreviations: AB = 1-amino-3-methyl-1-oxobutane; ADB = 1-amino-3,3-dimethyl-1-oxobutane; MDMB = methyl-3,3-dimethyl-butanoate; MMB = methyl-3-methyl-butanoate

Supplementary Table II: Efficacy (maximal reduction in G_{max}) and potency (IC_{50}) for the inhibition of the hERG channel and relative efficacy (E_{max}) and potency (EC_{50}) for the activation of the CB_1 receptor (where JWH-018 was used as the reference), calculated for the 36 SCRAs included in this study. SCRAs are organized by their four main structural components, as shown in Supplementary Table I. Efficacy and potency were not determined if no clear concentration–response relationship was observed or if IC_{50} values could not be estimated with defined confidence intervals. For fits without saturation within the tested concentration range (IC_{50} > 30 µM), results were considered reliable only if fit quality and parameter uncertainty met predefined criteria, specifically $R^2 > 0.8$ and $logIC_{50}$ span error < 1 (SI Table III).

SCRA	hERG channel				CB_1 receptor				Kv7.1/KCNE1 channel			
	Efficacy (max reduction in G_{max})		Potency (µM)		Efficacy (% JWH-018)		Potency (nM)		Efficacy (max reduction in G_{max})		Potency (µM)	
	G_{min}	SEM	IC_{50}	95% CI	E_{max}	SEM	EC_{50}	95% CI	G_{min}	SEM	IC_{50}	95% CI
APINACA	0.90*	ND	ND	ND	108	1.05	30.4	26.3-35.2	0.88*	ND	ND	ND
5F-APINACA	$0.00^{?}$	3.45	$106.7^{?}$	7.1-208.4	102	1.22	16.6	13.9-20.0	0	0.63	35.1	12.4-52.7
AB-FUBINACA	0.00	0.37	30.4	19.9-39.4	108	1.30	8.94	7.46-10.7	0.15	1.11	40.8	1.4-117.4
AB-CHMINACA	0.00	0.72	51.2	22.4-67.1	108	1.49	8.98	7.37-10.9	0.72*	ND	ND	ND
ADB-FUBINACA	0.25	0.14	13.7	4.8-30.4	106	1.23	4.97	4.13-6.02	0.81*	ND	ND	ND
ADB-CHMINACA	0.13	0.19	11.3	3.1-23.3	107	0.68	3.87	3.21-4.58	0.93*	ND	ND	ND
ADB-4en-PINACA	0.52	0.067	9.10	4.1-22.4	117	1.77	17.4	14.0-21.6	1.13*	ND	ND	ND
ADB-HEXINACA	0.037	0.19	15.8	7.3-22.1	117	1.21	39.4	34.6-44.8	0.41	0.11	1.99	0.1-9.7
Benzyl-4CN-BUTINACA	0.00	0.16	17.3	12.2-21	109	1.74	408	344-484	0.73*	ND	ND	ND
5F-Cumyl-PINACA	0.00	0.15	15.5	11.4-19.2	112	1.13	2.23	1.80-2.59	0.13	0.88	43.5	6.3-89.6
Cumyl-TsINACA	0.94*	ND	ND	ND	106	1.58	166	139-199	1.07*	ND	ND	ND
MDMB-FUBINACA	0.25	0.16	10.2	3.2-27.7	103	1.26	0.795	0.629-1.00	0.59*	ND	ND	ND
MDMB-CHMINACA	0.60	0.10	12.4	4.4-52.5	106	0.94	1.40	1.20-1.64	0.52*	ND	ND	ND
5F-MDMB-PINACA	0.00	0.11	9.0	4.8-9.9	131	1.62	8.99	7.50-10.8	0	1.65	71.4	24.7-113.5
MDMB-4en-PINACA	0.27	0.18	14.6	4.7-36.5	109	1.06	16.7	14.2-18.8	0.57*	ND	ND	ND
THJ-2201	0.73	0.037	2.04	0.7-5.5	92.8	1.17	45.6	28.4-54.2	0.59	0.09	4.7	1-22.4
ADB-5'Br-INACA	0.70	0.15	24.5	4.7-188.1	111	1.88	2290	1960-2670	1.04*	ND	ND	ND
ADB-5'Br-BUTINACA	0.36	0.36	40.1	10.3-100.5	110	1.20	18.0	15.5-21.0	0.80*	ND	ND	ND
MDMB-5'Br-INACA	0.57	0.18	25.3	7.2-115.5	110	1.26	849	759-949	0.74*	ND	ND	ND
MDMB-5'Br-BUTINACA	0.67	0.053	6.2	2.1-20.3	114	1.69	42.4	35.3-51.0	0.60*	ND	ND	ND
AB-CHMICA	0.64	0.051	8.2	3.4-21.8	110	0.92	20.1	17.7-22.7	0.46	0.18	16.2	1.9-67.6

SCRA	hERG channel				CB_1 receptor				$K_V7.1$/KCNE1 channel			
	Efficacy (max reduction in G_{max})		Potency (µM)		Efficacy (% JWH-018)		Potency (nM)		Efficacy (max reduction in G_{max})		Potency (µM)	
	G_{min}	SEM	IC_{50}	95% CI	E_{max}	SEM	EC_{50}	95% CI	G_{min}	SEM	IC_{50}	95% CI
ADB-CHMICA	0.48	0.10	5.2	1.2-21.9	110	0.41	15.7	14.5-17.0	0.4	0.19	13.4	2.2-50.9
4F-MDMB-BUTICA	0.35	0.13	26.2	13.4-57.1	115	1.34	16.7	14.3-19.6	0.87*	ND	ND	ND
MDMB-CHMICA	0.55	0.051	3.2	1.2-8.3	107	0.69	3.07	2.72-3.46	0.78*	ND	ND	ND
MMB-CHMICA	0.75	0.041	2.6	0.7-7.9	109	0.91	8.76	7.69-9.97	0.83	ND	ND	ND
JWH-018	0.57	0.21	17.3	3.4-102.5	100	0.41	35.0	29.8-41.2	1.01*	ND	ND	ND
AM-2201	0.38	0.042	1.3	0.8-2.1	99.7	1.62	25.8	20.1-33.2	0.92*	ND	ND	ND
JWH-122	0.73	0.16	9.4	0.3-214	105	1.34	8.29	6.73-10.2	0.87*	ND	ND	ND
JWH-210	0.89*	ND	ND	ND	110	1.58	10.1	8.11-12.6	0	4.61	141.5	12.7-278.7
UR-144	0.94*	ND	ND	ND	101	1.73	414	342-503	0.92*	ND	ND	ND
XLR-11	0.54	0.13	20.4	7-80.8	105	0.96	260	234-288	0.87*	ND	ND	ND
BZO-POXIZID	0.79*	ND	ND	ND	108	2.28	349	282-432	0.83*	ND	ND	ND
BZO-4en-POXIZID	0.64	0.054	3.9	1.6-9.4	112	3.32	1700	1320-2210	0.79*	ND	ND	ND
5F-BZO-POXIZID	0.29	0.037	0.9	0.6-1.2	113	2.51	193	147-252	0.83*	ND	ND	ND
BZO-HEXOXIZID	0.00[?]	1.53	63.6[?]	12.5-106.6	84.3	3.15	5130	3950-6670	0.44	0.12	3.0	0.3-20.7
JWH-030	0.00	0.28	23.1	16.1-29.7	76.5	0.80	291	257-328	0	0.2	12.2	8.3-16.8

Efficacy (maximal reduction in G_{max}) is in the table indicated as G_{min}, where 1 indicates no reduction of G_{max} and 0 indicates complete reduction of G_{max} (i.e., no current remaining). ND, not determined (values could not be reliably estimated). [?] Unreliable values. * Greatest efficacy achieved with a concentration between 10 and 30 µM. n = 3-13 for hERG and n = 3-9 for $K_V7.1$/KCNE1. For CB_1, Results are represented as receptor activity of JWH-018 (%) derived from a minimum of three independent experiments (n ≥ 3), run in triplicate (except for JWH-030, for which there were only two independent experiments, n = 2).

Supplementary Table III. Quality control (QC) assessment for estimated values for SCRAs with extrapolated IC_{50}-values (> 30 µM) on hERG using APC. G_{min}, max reduction in G_{max} estimated from the fit; SEM, standard error of the estimate; IC_{50}, half-maximal inhibitory concentration estimated from the fit; 95% CI, asymmetrical confidence interval for IC_{50}; $logIC_{50}$ span error, width of the confidence interval in log space (difference between upper and lower bounds); R^2, goodness-of-fit. For SCRAs with extrapolated IC_{50}-values, estimated values were considered reliable only if fit quality and parameter uncertainty met predefined criteria ($R^2 > 0.8$ and $logIC_{50}$ span error < 1).

SCRA	G_{min}	SEM	IC_{50}	95% CI	$logIC_{50}$ span error	R^2	QC comment for G_{min} and IC_{50}
5F-APINACA	0.00	3.45	106.7	7.056 to 208.4	1.47	0.2174	Unreliable ($logIC_{50}$ span error > 1; R2 < 0.8)
AB-CHMINACA	0.00	0.72	51.2	22.38 to 67.11	0.48	0.8346	Reliable
ADB-5Br-BUTINACA	0.36	0.36	40.1	10.27 to 100.5	0.99	0.8042	Reliable
BZO-HEXOXIZID	0.00	1.53	63.6	12.45 to 106.6	0.93	0.4628	Unreliable (R2 < 0.8)

Supplementary Table IV. SAR analysis for impact of core moiety on CB_1 receptor efficacy (E_{max}) and potency (EC_{50}). P values for compound-to-compound comparisons are from Brown-Forsythe and Welch ANOVA tests. P values for overall comparisons are from two-tailed paired t-tests. Trend denotes overall effects that are not significant. but all go in the same direction.

	Comparisons		Efficacy (E_{max})			Potency ($logEC_{50}$)		
	**Compound 1**	**Compound 2**	**Mean Diff**	**P value**	**Significant?**	**Mean Diff**	**P value**	**Significant?**
**Indole vs. Indazole**	AB-CHMICA	AB-CHMINACA	2.081	0.9247	No	0.3489	0.0216	Yes
	ADB-CHMICA	ADB-CHMINACA	3.284	0.1469	No	0.6109	0.0058	Yes
	MDMB-CHMICA	MDMB-CHMINACA	0.6356	0.9998	No	0.3405	0.0150	Yes
	AM-2201	THJ-2201	6.900	0.0641	No	-0.2500	0.0464	Yes
**Overall comparison** (mean Diff ± SEM)			**-3.225 ± 1.291**	**0.0879**	**No (trend)**	**0.2626 ± 0.1820**	**0.2448**	**No**

Supplementary Table V. SAR analysis for impact of head moiety on CB_1 receptor efficacy (E_{max}) and potency (EC_{50}). P values for compound-to-compound comparisons are from Brown-Forsythe and Welch ANOVA tests. P values for overall comparisons are from two-tailed paired t-tests. Trend denotes overall effects that are not significant. but all go in the same direction.

	Comparisons		Efficacy (E_{max})			Potency ($logEC_{50}$)		
	**Compound 1**	**Compound 2**	**Mean Diff**	**P value**	**Significant?**	**Mean Diff**	**P value**	**Significant?**
**AB vs. ADB**	AB-FUBINACA	ADB-FUBINACA	2.638	0.8301	No	0.2541	0.1145	No
	AB-CHMINACA	ADB-CHMINACA	1.015	0.9991	No	0.3663	0.0395	Yes
	AB-CHMICA	ADB-CHMICA	-0.1872	>0.9999	No	0.1043	0.2688	No
**Overall comparison** (mean Diff ± SEM)			**-1.000 ± 0.5774**	**0.2254**	**No**	**0.2416 ± 0.07589**	**0.0861**	**No (trend)**

**AB vs. MDMB**	AB-FUBINACA	MDMB-FUBINACA	5.054	0.3044	No	1.051	0.0011	Yes
	AB-CHMINACA	MDMB-CHMINACA	1.170	0.9983	No	0.8071	0.0017	Yes
	AB-CHMICA	MDMB-CHMICA	2.616	0.4704	No	0.8155	0.0003	Yes
**Overall comparison** (mean Diff ± SEM)			**3.333 ± 0.8819**	**0.0634**	**No (trend)**	**0.8912 ± 0.07993**	**0.0079**	**Yes**

**ADB vs. MDMB**	ADB-FUBINACA	MDMB-FUBINACA	2.416	0.8719	No	0.7968	0.0033	Yes
	ADB-CHMINACA	MDMB-CHMINACA	0.1542	>0.9999	No	0.4408	0.0120	Yes
	ADB-4en-PINACA	MDMB-4en-PINACA	8.179	0.1623	No	0.0200	>0.9999	No
	ADB-5'Br-INACA	MDMB-5'Br-INACA	1.044	>0.9999	No	0.430	0.0049	Yes
	ADB-5'Br-BUTINACA	MDMB-5'Br-BUTINACA	-3.822	0.6589	No	-0.3675	0.0251	Yes
	ADB-CHMICA	MDMB-CHMICA	2.803	0.2178	No	0.7111	0.0013	Yes
**Overall comparison** (mean Diff ± SEM)			**2.000 ± 1.592**	**0.2644**	**No**	**0.3385 ± 0.1796**	**0.1181**	**No**

Supplementary Table VI. SAR analysis for impact of tail moiety on CB_1 receptor efficacy (E_{max}) and potency (EC_{50}). P values for compound-to-compound comparisons are from Brown-Forsythe and Welch ANOVA tests. P values for overall comparisons are from two-tailed paired t-tests.

	**Comparisons**		**Efficacy (E_{max})**			**Potency ($logEC_{50}$)**		
	**Compound 1**	**Compound 2**	**Mean Diff**	**P value**	**Significant?**	**Mean Diff**	**P value**	**Significant?**
CHM vs. FUB	AB-CHMINACA	AB-FUBINACA	-0.9264	0.9488	No	0.00227	>0.9999	No
	ADB-CHMINACA	ADB-FUBINACA	0.6963	0.9923	No	-0.1099	0.4982	No
	MDMB-CHMINACA	MDMB-FUBINACA	2.958	0.4462	No	0.2461	0.1342	No
**Overall comparison** (mean Diff ± SEM)			**1.333 ± 0.8819**	**0.2697**	**No**	**0.04615 ± 0.1051**	**0.7035**	**No**
Pentyl vs. 5F-Pentyl	BZO-POXIZID	5F-BZO-POXIZID	-5.298	0.5935	No	0.2500	0.1565	No
	UR-144	XLR-11	-4.439	0.2434	No	0.2035	0.0778	No
	APINACA	5F-APINACA	5.997	0.0507	No	0.09912	0.4648	No
**Overall comparison** (mean Diff ± SEM)			**-0.9000 ± 3.612**	**0.8265**	**No**	**0.1842 ± 0.04461**	**0.0539**	**No (trend)**

Supplementary Table VII. Summary of experimental conditions for electrophysiology experiments.

Construct	Approach	Cell format	Dosing mode	Settling time	Voltage ladder protocol	Temperature
hERG	APC	Single-cell patch	Liquid exchange via microfluidic channels; cumulatively	60 s	Protocol #1 (see below)	22°C
hERG	MPC	Single-cell patch	Pressure-driven directed flow; cumulatively	30 s	Protocol #2 (see below)	22-24°C
Kv7.1/KCNE1	APC	Population patch (up to 10 cells/well)	Liquid exchange via microfluidic channels; cumulatively	60 s	Protocol #3 (see below)	22°C

Abbreviations: APC: automated patch clamp; MPC: manual patch clamp.

Protocol #1: Holding voltage = –80 mV; voltage steps = –60 to +60 mV in 10 mV steps (5 s each); tail voltage = –50 mV (2 s). Note: 50 ms pre-pulse to –90 mV for ohmic leak compensation.

Protocol #2: Holding voltage = –80 mV; voltage steps = –60 to +50 mV in 10 mV steps (5 s each); tail voltage = –50 mV (2 s). Note: 50 ms pre-pulse to –90 mV for ohmic leak compensation.

Protocol #3: Holding voltage = –90 mV; voltage steps = –100 to +40 mV in 10 mV steps (4 s each); tail voltage = –20 mV (2 s). Note: 540 ms pre-pulse to –100 mV for ohmic leak compensation.
